# Unlocking Genetic Profiles with a Programmable DNA‐Powered Decoding Circuit

**DOI:** 10.1002/advs.202206343

**Published:** 2023-04-28

**Authors:** Junlan Liu, Chao Zhang, Jinxing Song, Qing Zhang, Rongjun Zhang, Mingzhi Zhang, Da Han, Weihong Tan

**Affiliations:** ^1^ Institute of Molecular Medicine (IMM) Renji Hospital School of Medicine and College of Chemistry and Chemical Engineering Shanghai Jiao Tong University Shanghai 200240 China; ^2^ The Key Laboratory of Zhejiang Province for Aptamers and Theranostics, Zhejiang Cancer Hospital Hangzhou Institute of Medicine (HIM) Chinese Academy of Sciences Hangzhou Zhejiang 310022 China; ^3^ Molecular Science and Biomedicine Laboratory (MBL) State Key Laboratory of Chemo/Biosensing and Chemometrics College of Chemistry and Chemical Engineering College of Biology Aptamer Engineering Center of Hunan Province Hunan University Changsha Hunan 410082 China

**Keywords:** DNA computation, genetic interpretation, genotype‐phenotype translation, molecular engineering, precision medicine

## Abstract

Human genetic architecture provides remarkable insights into disease risk prediction and personalized medication. Advances in genomics have boosted the fine‐mapping of disease‐associated genetic variants across human genome. In healthcare practice, interpreting intricate genetic profiles into actionable medical decisions can improve health outcomes but remains challenging. Here an intelligent genetic decoder is engineered with programmable DNA computation to automate clinical analyses and interpretations. The DNA‐based decoder recognizes multiplex genetic information by one‐pot ligase‐dependent reactions and interprets implicit genetic profiles into explicit decision reports. It is shown that the DNA decoder implements intended computation on genetic profiles and outputs a corresponding answer within hours. Effectiveness in 30 human genomic samples is validated and it is shown that it achieves desirable performance on the interpretation of *CYP2C19* genetic profiles into drug responses, with accuracy equivalent to that of Sanger sequencing. Circuit modules of the DNA decoder can also be readily reprogrammed to interpret another pharmacogenetics genes, provide drug dosing recommendations, and implement reliable molecular calculation of polygenic risk score (PRS) and PRS‐informed cancer risk assessment. The DNA‐powered intelligent decoder provides a general solution to the translation of complex genetic profiles into actionable healthcare decisions and will facilitate personalized healthcare in primary care.

## Graphical abstract

An intelligent genetic decoder powered by DNA computation can interpret complex human genetic profiles into straightforward medical decision reports in a test tube. It eliminates laborious manual analyses and technical threshold raised by in silico computation and serves as a promising tool to facilitate personalized medication, genetic counseling, and cancer risk prediction in primary care.

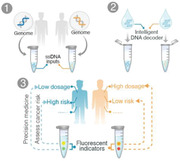

## 1 Introduction

Genetic variations contribute dramatically to the inter‐individual phenotypic variability, ranging from eye color to disease susceptibility.^[^ ^1^ ^]^ Over the past decade, tremendous advances in genomic technologies have revolutionized the ability of genome‐wide association studies (GWAS) to pinpoint human genetic variations implicated in complex diseases, such as cancer and type 2 diabetes (T2D).^[^ ^2^ ^]^ Discoveries of numerous risk variants not only shed light on the pathogenesis of complex diseases but also enable the screening of individuals at high risk of complex diseases,^[^ ^3^ ^]^ providing opportunities for prophylactic actions, and guiding precision medicine.^[^ ^4^ ^]^ In healthcare practice, however, there are still huge gaps to be bridged between genetic discoveries and their actual uses. One major obstacle to filling the gap lies in the translation of individual genetic profiles into actionable healthcare decisions. In most cases, associations between genetic variants and phenotypes are not as simple as those in exceptionally rare monogenic disorders, which are genetically controlled by a single locus (e.g., a single base‐pair substitution).^[^ ^5^ ^]^ Most disorders are determined by the overall effect of numerous genetic variations with varying degrees of contributions.^[^ ^6^ ^]^ This complex genetic architecture makes it clinically challenging to translate implicit genetic information into explicit healthcare decisions.^[^ ^7^ ^]^ Current genotyping techniques acquire genetic data by specialized instrumentation and data analysis pipeline,^[^ ^8^ ^]^ but of particular note, it is the phenotype predicted from multiplex genetic data that offers health benefits. In the context of precision medicine, for example, clinical prescribing actions rely on individual drug responses predicted from multiple genetic determinants of drug efficacy and/or toxicity.^[^ ^4c^ ^]^ Accordingly, in silico computational tools,^[^ ^9^ ^]^ along with documented guidelines^[^ ^10^ ^]^ and expert data interpretation,^[^ ^11^ ^]^ are quite necessary to finalize the workflow of traditional genetic services but are inconsistently implemented across clinical laboratories,^[^ ^12^ ^]^ thereby discouraging wide‐scale incorporation of genetics‐directed personalized healthcare into routine clinical practice.

A theoretically promising approach for intelligent genotype‐phenotype interpretation is DNA‐based molecular computing circuitry. Thus far, DNA has been extensively used in molecular engineering as a versatile, programmable, and biocompatible building block.^[^ ^13^ ^]^ Furthermore, the thermodynamics and kinetics of DNA hybridization and strand displacement have been thoroughly characterized,^[^ ^14^ ^]^ making it possible to tailor DNA circuits for diverse computational tasks, such as Boolean logic operations,^[^ ^15^ ^]^ basic arithmetic computation,^[^ ^16^ ^]^ and even neural network mimicry.^[^ ^17^ ^]^ More importantly, DNA is much flexible to interface with biological systems, opening up many possibilities for DNA computation in clinical diagnostics,^[^ ^18^ ^]^ bio‐imaging,^[^ ^19^ ^]^ and therapeutics.^[^ ^20^ ^]^ Therefore, it can be anticipated to engineer an intelligent DNA decoding circuit able to recognize and interpret multiplex genetic profiles into actionable healthcare decisions in a “tube” without in silico computation and expert data interpretation.

Herein, we established a general framework built on a programmable DNA decoding circuit to facilitate the translation of complex genetic profiles into actionable medical decisions. To make genetic information recognizable, we developed a ligase‐based signal transforming strategy to accurately and conveniently convert multiplex allelic information into a battery of single‐stranded DNA uniquely mapped to DNA gates in the DNA decoding circuit by a one‐pot ligase‐dependent reaction (LDR). This strategy leverages the allele‐discriminating power of thermostable ligase, thus circumventing mis‐extension errors often encountered in polymerase‐based genotyping methods, such as TaqMan genotyping assays and amplification refractory mutation system polymerase chain reaction (PCR).^[^ ^21^ ^]^ The DNA decoding circuit is a highly modular and programmable arithmetic circuit with the ability to “acquire” genetic knowledge and then translate various sets of genetic binary codes (i.e., the presence of an allele is coded as 1 and absence as 0) into actionable healthcare decisions (indicated by fluorescent signals) according to pre‐defined programs with modest instrument requirements. We show that the DNA decoding circuit implements expected computation for varying combinations of synthetic inputs and when tested in human genomic DNA samples, still achieves desirable performance on the interpretation of “genetic codes” of *CYP2C19*, a pharmacogenetics (PGx) gene, into drug responses, with accuracy equivalent to that of Sanger sequencing. We also demonstrate that circuit layouts can be readily rewired for other healthcare purposes, such as interpreting pharmacogenetics tests, providing drug dosing recommendations, and implementing reliable molecular calculation of polygenic risk score (PRS) as well as PRS‐informed disease risk assessment.

## 2 Results

### 2.1 Overview of the DNA‐Powered Intelligent Genetic Decoder

**Figure 1:**
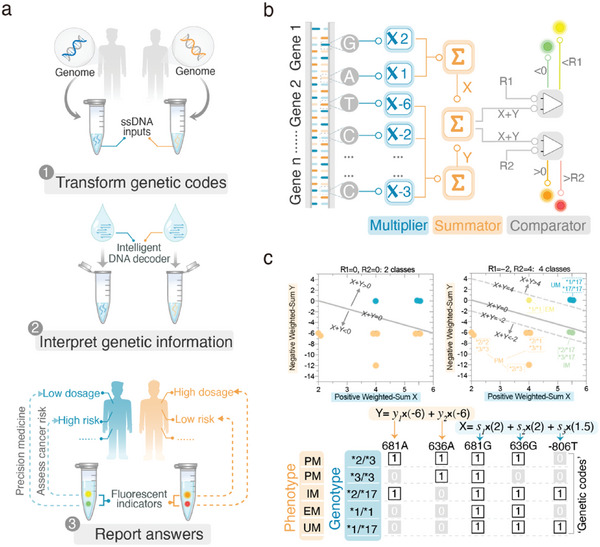
The working scheme of an intelligent DNA‐based genetic decoder. a) Overview of a decoding workflow with an intelligent DNA computing circuit. In a “sample in, answer out” manner, the DNA decoding circuit can recognize the “genetic codes” of a human genomic sample and automatically figure out the most probable answer (e.g., drug dosing recommendation or cancer risk) according to “acquired” genetic knowledge, which is customizable to varying healthcare purposes. b) Three essential modules in the DNA decoding circuit. The DNA decoding circuit interprets “genetic codes” by performing arithmetic operations over multiplex alleles with three modules: multiplier and summator for weighted‐sum calculation, and comparators for numeric comparison of weighted‐sum. Each allele at a specific genetic locus is uniquely mapped to a multiplier in the circuit, which is activated only by the target allele. c) Linearly separable phenotypic classes in the 2D weighted‐sum space. Data shown were four distinct phenotypic classes of drug responses related to *CYP2C19* polymorphisms. Each point on the two scatter plots represents one *CYP2C19* genotype and identical phenotypic classes share the same color. The “genetic codes” are shown in such a way that present alleles are marked as “1” while absent allele are marked as “0”. Genotypes are shown as star (*) alleles. PM: poor metabolizers; IM: intermediate metabolizers; EM: extensive metabolizers; UM: ultrarapid metabolizers. A detailed genotype‐phenotype correlation is listed in Table S1, Supporting Information.

A DNA‐based decoding circuit with previously acquired genetics knowledge can recognize multiplex genetic profiles and then output the most probable phenotype of an individual in a tube, thus it can provide users with explicit reports in a “sample in, answer out” fashion (**Figure** **1**a). Also, circuit programs are reconfigurable for varying healthcare purposes, such as dose recommendations and disease risk assessment.

The underlying principle is a series of molecular arithmetic operations over a battery of effect alleles with established roles in inter‐individual phenotypic variabilities (Figure 1b). At first, all alleles are multiplied individually by their respective weights (Figure 1b), which depend on the contribution to phenotype expression. Specifically, a loss‐of‐function allele may take a negative weight whilst a gain‐of‐function allele may take a positive weight. All weighted alleles then add up to an overall score (Figure 1b), which varies with distinct phenotypic classes. Accordingly, comparators are incorporated to distinguish different levels of weighted‐sum and output fluorescent signals (Figure 1b).

To program a decoding circuit, a critical step is to learn genetic features underlying diverse phenotypes, that is, to look for an optimal array of multiplicative coefficients assigned to each allele. An optimal coefficient array should make all output classes separable in the weighted‐sum space such that the classification task is formulated as a set of mathematical operations. To clarify it, we take four phenotypic classes of drug responses caused by *CYP2C19* single nucleotide polymorphisms (SNPs) as a demonstration.^[^ ^22^ ^]^ Since each SNP (or exactly biallelic SNP) gives rise to two alternative alleles within a specific locus, multiple SNPs across different loci can lead to various combinations of all alternative alleles, which we refer to as “genetic codes” (Figure 1c). All genotypes were denoted by the star (*) alleles according to the star allele nomenclature, where each number represents a specific allele with altered functionality caused by a single variant or a group of genetic variations.^[^ ^23^ ^]^ For each *CYP2C19* genotype, there is a particular set of “genetic codes” encoded by six possible *CYP2C19* alleles (Table S1, Supporting Information). For functional classification, individuals with different genotypes are generally categorized into four phenotypic categories: poor (PM), intermediate (IM), extensive (EM), and ultrarapid (UM) metabolizers. Genotypes of the same phenotypic class, though holding distinct “genetic codes”, should well separate from other classes in the 2D weighted‐sum space (Figure 1c). Accordingly, multiplex genetic profiles were reduced to two dimensions (*X* vs *Y*), and the classification of multiple phenotypic classes thus can be achieved by comparing weighted‐sum numerically with reference values (Figure 1c). To facilitate the learning process of allelic features, a MATLAB script was written to automatically look for fitted coefficient arrays for a given phenotype‐genotype dataset (Note S1, Supporting Information).

### 2.2 Conversion from Genetic Profiles into Recognizable Inputs

**Figure 2:**
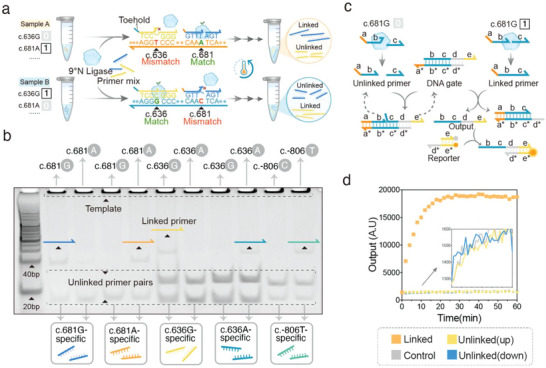
Conversion from genetic profiles into recognizable ssDNA inputs. a) Schematic illustration of ligase‐dependent reaction (LDR). The upstream primer with a particular toehold domain at its 5’ end is allele‐specific, while the downstream phosphorylated primer is shared by all alleles at the same genetic locus. If the target allele is present within the given sample (indicated by “1”), both primers could be joined together by the thermostable 9°N ligase at each thermal cycle after simultaneously hybridizing to the target allele. If the target allele is absent (indicated by “0”), the ligation between the primer pair is prevented by the mismatched nucleotide at the gap. For simplicity, only the template strand for ligation was shown. b) Cross‐validation of LDR specificity. Five primer pairs designated for *CYP2C19* alleles were used to validate LDR specificity. Synthetic plasmids with indicated allelic sequences were used as the template. For each primer pair, successfully linked primers were only detectable in the context of the targeted template, whereas none of the ligated primers were detected in the context of non‐targeted template. The first lane was loaded with 5 µL of 20‐bp marker. Primer sequences are detailed in Table S2, Supporting Information. c) The DNA strand‐displacement reactions between a DNA gate and linked/unlinked primers. The linked primer is thermodynamically favorable to displacing an output strand from the DNA gate, whereas the unlinked primer is thermodynamically unfavorable for that. d) Experimental validation of the ability of linked (10 nm synthetic oligonucleotides) and the disability of unlinked primer (100 nm synthetic oligonucleotides) to displace output strands. The *CYP2C19* c.636A‐specific primer pair was used. The experiment was performed at 37 °C. Unless specified otherwise, DNA strands were all denoted by lines, with their color indicating distinct domains and ticks marking its 3’ ends.

Converting genetic profiles into recognizable inputs is an essential prerequisite in attempts to engineer molecular genetic decoders. To this end, we developed a thermostable ligase‐based transforming strategy to accurately transform multiplex genetic information into DNA strands with unique sequences through a one‐pot ligase‐dependent reaction (LDR) (**Figure** **2**a). LDR achieves precise allelic discrimination by the thermostable 9°N™ ligase that can covalently join the end of two immediately adjacent and perfectly matched primers following binding to the target template at elevated temperatures, whereas any variations (e.g., SNPs, Insertions, and Deletions) at the junction can prevent them from being successfully ligated (Figure 2a). Each allele has an upstream allele‐specific primer with a particular toehold domain at its 5’ end, and a phosphorylated downstream primer (Figure 2a). When the target allele is present, both primers will hybridize with the target allele and be joined together by ligase, generating a linked primer at each thermal cycle (Figure 2a). However, there is none of linked primers in the absence of the target allele (Figure 2a). The allele‐discriminating power of LDR was cross‐validated using five primer pairs designed for five *CYP2C19* alleles (see Table S2, Supporting Information for detailed sequences). Five synthetic plasmids carrying a 4.81 kb gene fragment of corresponding alleles were used as the template of LDR. It demonstrated that linked primers were detectable only in the context of targeted allelic templates, whereas none of linked primers were detected in the context of non‐targeted allelic templates (Figure 2b). Next, we used ImageJ software to quantify band intensities of linked and unlinked primers on the gel image and approximated their concentrations based on the initial total concentration,^[^ ^24^ ^]^ revealing that the ligation efficiency ranged from 35% to 64% and unspecific ligation events were no more than 2.1% (Table S3, Supporting Information). Overall, it demonstrates that LDR maintains a high selectivity against point mutations while giving a desirable yield of ligated products.

As the toehold domain on the allele‐specific primer is perfectly complementary to that of an allele‐specific DNA arithmetic gate, successfully linked allele‐specific primers can initiate a strand displacement reaction with allele‐specific DNA gate and displace an output strand (Figure 2c). Instead, both unlinked primers are thermodynamically unfavorable to displacing the output (Figure 2c). Experimentally characterized kinetics behaviors of five LDR primer pairs verified that only linked primer can initiate the strand displacement reaction, yet unlinked primers were observed to be unable to complete the strand displacement (Figure 2d) (Figure S1, Supporting Information). Characterization of reaction products by gel electrophoresis also shows that only linked primer is able to displace the output strand from the DNA gate and hybridize with the bottom strand of the DNA gate (Figure S2, Supporting Information), further verifying this proposed reaction pathway. We also demonstrated that the ligase‐based method could reliably transform DNA methylation into unique circular ssDNA products when combined with molecular inversion probe (MIP). MIP is essentially a single‐stranded oligonucleotide, with two ends adjacent and complementary to flanking sequences of target CpG, and a middle domain for downstream mathematical operations. Both ends of the probe targeting the methylation sites is complementary to the methylated template converted by sodium sulfite (Figure S3a, Supporting Information). We designed a pair of MIPs for Cg16655791 and experimentally characterized that linear MIPs will be circularized by thermostable ligase only in the context of correct template, while mismatches at the joint will prevent it from being ligated. Exonuclease I and exonuclease III will remove linear probes and double‐stranded template respectively, leaving only circular probes for downstream molecular operations (Figure S3b, Supporting Information).

### 2.3 Molecular Arithmetic Operations over Genetic Profiles

**Figure 3:**
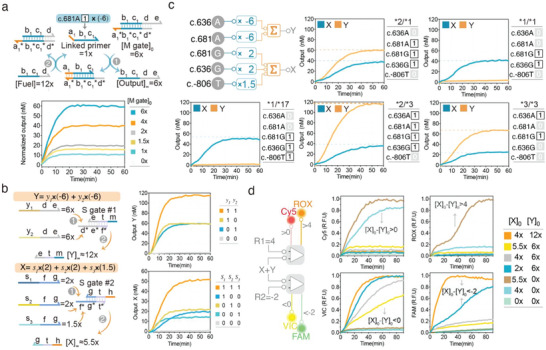
Molecular arithmetic operations over distinct genotypes. a) Schematic illustration and experimental validation of molecular multiplication. The multiplicative coefficient is determined by the initial concentration of the multiplication gate (M gate). The concentration of fuel exceeds that of the M gate. Linked primer (synthetic DNA) was consistently given at 1× (10 nm), while output concentrations varied with different concentrations of M gate. b) Schematic illustration and experimental validation of molecular summation. Excessive summation gate (S gate) can integrate weighted outputs displaced from distinct upstream M gates into a common output strand. S gate #1 integrates all negatively weighted species into output *Y*, while S gate #2 integrates all positively weighted species into output *X*. For all test cases, 1 or 0 corresponded to the presence or absence of an input strand (synthetic linked primer), respectively. c) Parallel computation of positive and negative weighted‐sum for distinct *CYP2C19* genotypes (indicated by star alleles). “Genetic codes” of each genotype were indicated. A multiplicative coefficient was assigned to each allele. LDR products (using synthetic plasmids as templates) were used as inputs for the arithmetic module of the DNA decoding circuit. d) Experimental validation of weighted‐sum comparators. Serial combinations of *X* and *Y* strands at indicated initial concentrations were used as inputs. The initial concentration of *X* ([*X*]_0_) minus that of *Y* equals the final weighted‐sum ([*X*]_0_−[*Y*]_0_). VIC and Cy5 signals signified a weighted‐sum below and above zero, respectively. FAM and ROX signals signified a weighted‐sum below “−2” and above “4”, respectively. The data shown were normalized relative fluorescence units (R.F.U). All experiments were performed at 37 °C. 1× = 10 nm for all cases.

The general scheme for molecular mathematic implementation is based on the toehold‐mediated strand‐displacement reaction network between distinct DNA arithmetic gates at predetermined concentrations. Specifically, multiplicative computation was powered by a panel of “seesaw catalyst”‐based multiplication gates designated for each allele (Figure S4a, Supporting Information). Though concentrations of linked primers may vary with different alleles, weighted outputs for each allele can be definitive by fixing the concentration of the multiplication gate in that a small number of input species can activate catalytic strand‐displacement reaction.^[^ ^17b^ ^]^ The multiplication gate has a double‐stranded central domain and two single‐stranded flanking domains, of which one side is the toehold that can be recognized by a linked primer to initiate branch migration, while the other shares identical sequences with the branch migration domain of downstream summation gates (**Figure** **3**a). After hybridizing with the toehold on the multiplication gate, the linked primer will displace an output strand and activate the other toehold (d*) simultaneously (Figure 3a). Excessive fuel strands will recognize the newly uncovered toehold and initiate another strand‐displacement cycle to free up the linked primer, thereby allowing linked primers to be reused over multiple cycles (Figure 3a). Outputs of DNA multiplication gates at user‐defined molar concentrations were demonstrated to be completely displaced by corresponding linked primers even at six‐times lower quantity (Figure 3a) (Figure S5, Supporting Information).

The summation of weighted species displaced from multiplication gates was realized by two partially double‐stranded DNA summation gates, which respectively convert all positively weighted species into identical output *X* and negatively‐weighted species into *Y* (Figure 3b). With a specific toehold domain, any weighted species with complementary sequences can initiate branch migration on summation gate (Figure 3b). Each summation gate was individually tested with synthetic inputs of multiplication gates, demonstrating that the summation gate could exactly transform weighted species displaced from distinct upstream multiplication gates into identical outputs (Figure 3b).

We next validated whether the two arithmetic gates could accurately compute weighted‐sum for different *CYP2C19* genotypes. With a special panel of “genetic codes,” each genotype would have a particular combination of linked primers in LDR products. Using serial combinations of synthetic linked primers as inputs, the circuit yielded desired concentrations of weighted‐sum species *X* and *Y* for all test cases (Figure S4b, Supporting Information), indicating that allele‐specific multiplication gates could work in parallel to compute weighted species. When input species were changed from synthetic DNA strands to products of LDR (plasmid templates were prepared according to the specific allele status of each genotype, as shown in Table S4, Supporting Information), the circuit still outputted desired concentrations of weighted‐sum *X* and *Y* for all tested *CYP2C19* genotypes (Figure 3c) (Figure S6, Supporting Information), indicating that the circuit could recognize transformed “genetic codes” and perform arithmetic operations over the mixture of ligase‐mediated linked primers, whose components may vary with genotypes. However, it still requires a subtraction gate to compute “*X+Y*” in that species *Y* represents a negative weighted‐sum. This can be achieved by a cooperative hybridization scheme,^[^ ^25^ ^]^ whereby stoichiometrically equivalent amounts of species *X* and *Y* can simultaneously hybridize with the subtraction gate and irreversibly annihilate each other only if both species are present. To realize molecular subtractive operations, we additionally designed two DNA transformation gates (Figure S7a, Supporting Information), whose strand‐displacement kinetics were programmed to be much slower than the subtraction gate by cutting 2 nucleotides from its toehold domains (the length and sequence of the toehold domain are known to significantly influence the kinetics of strand displacement^[^ ^14a^ ^]^). Accordingly, the weighted‐sum with a lower absolute quantity will be exhausted and the leftover of the higher weighted‐sum species will sequentially hybridize with and displace an output strand (H1 or H2) from a transformation gate stoichiometrically (Figure S7a, Supporting Information). We used 12 combinations of weighted‐sum *X* and *Y* at initial concentrations ranging from 0 to 120 nm to demonstrate the subtraction scheme and observed that the circuit behavior generally agree with the proposed design: if the initial concentration of *Y* is higher than that of *X*, then the concentration of the H1 strand is approximate to the leftover amount of *Y* ([H1]_∞_≈[*Y*]_0_−[*X*]_0_); when the initial concentration of *X* is higher that of *Y*, the concentration of H2 strand is approximate to the leftover amount of *X* ([H2]_∞_≈[*X*]_0_−[*Y*]_0_) (Figure S7b, Supporting Information). In this way, weighted‐sum species computed by upstream arithmetic gates are transformed into different concentrations of species H1 or H2 that can be processed by downstream comparators.

To implement numeric comparison with DNA molecules, we took advantage of two threshold gates in conjunction with four catalytic fluorescence reporting systems, among which VIC and Cy5 reporters were respectively programmed to indicate the presence of H1 (H1 > 0) and H2 (H2 > 0), while FAM and ROX reporters were expected to output fluorescence only if H1 and H2 exceed a user‐defined threshold, respectively (Figure S8a, Supporting Information). The species H1 and H2 were programmed to preferentially hybridize with threshold gates by supplementing the toehold on threshold gates with extra nucleotides, thereby rendering strand displacement reactions of threshold gates much faster than those of restoration gates (Figure S8a, Supporting Information). We experimentally tested the thresholding effectiveness using varying concentrations of species H and observed that the circuit could trigger the desired fluorescent signal(s) for species H above the threshold, whereas undesired signals, though detectable, maintained a desirable OFF state in cases of species H below the threshold (Figure S8b, Supporting Information). Further, we verified the thresholding effectiveness of the comparator connected with a subtraction module using six distinct combinations of *X* and *Y* (Figure 3d). In agreement with the circuit design, significant VIC signals were only observed in instances of [*X*]_0_−[*Y*]_0_ < 0 ([H1] > 0), whilst Cy5 signals were observed only if [*X*]_0_−[*Y*]_0_ > 0 ([H2] > 0), whereas ROX and FAM signals remarkably increased only when [*X*]_0_−[*Y*]_0_ exceeds the predefined threshold (Figure 3d). Overall, these results suggest that the DNA‐based decoding circuit can recognize and compute genetic profiles of distinct genotypes and export desired fluorescent signals predefined for the phenotypic class of a genotype.

### 2.4 Evaluating the Performance of the Genetic Decoder on Human Genomic DNA Samples

**Figure 4:**
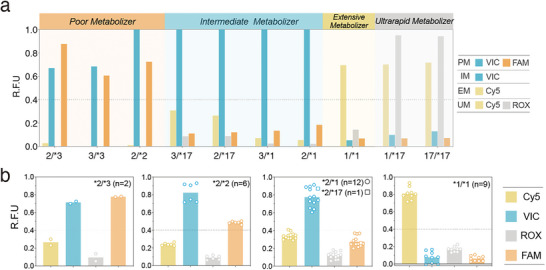
The performance of the DNA‐powered genetic decoder on human genomic DNA samples. a) Interpreting *CYP2C19* “genetic codes” into drug responses for synthetic plasmid samples. Two premixed plasmids carrying sequences corresponding to indicated *CYP2C19* diplotypes were used as the template of LDR. Phenotyping results were denoted by distinct fluorescent patterns. The dashed line indicates an R.F.U threshold of 0.4. b) Interpreting *CYP2C19* “genetic codes” into drug responses for 30 human genomic DNA samples. These samples were tested in different batches of experiments (1st batch: *n* = 2; 2nd batch: *n* = 6; 3rd batch: *n* = 15; 4th batch: *n* = 7). Genotypes of these samples were indicated by star alleles and the number of cases was shown in brackets. Note that only five highly prevalent genotypes were included. The sample whose *CYP2C19* genotype is *2/*17 was denoted by a square. Data shown were end‐point (2.5 h) relative fluorescence units (R.F.U) normalized by fluorescence from negative control (R.F.U = 0) and the standard initiator strand of each fluorescent reporter (R.F.U = 1). Bars denote mean RFUs.

To demonstrate the robustness and usefulness of the genetic decoder, we collected 30 distinct human genomic DNA samples whose *CYP2C19* genotypes have been validated using Sanger sequencing (Table S5, Supporting Information). Initially, we used synthetic inputs to test if the decoding circuit could yield expected fluorescent patterns for each phenotype. Though desired fluorescent patterns were observed for all phenotypic classes, undesired signals, particularly those of FAM and Cy5, remained detectable (Figure S9, Supporting Information). To reproducibly demarcate ON/OFF state, an RFU threshold of 0.4 was defined according to FAM/Cy5 RFU of simulated intermediate metabolizer samples. Further, we evaluated the performance of the entire workflow using binary combinations of synthetic plasmids as simulated samples. As observed, all genotypes could be assigned to the expected phenotype based on their fluorescent outputs (**Figure** **4**a). Finally, we assessed the performance of the DNA‐powered genetic decoder on 30 human genomic DNA samples. All samples were first subjected to pre‐amplification owing to the extremely low copy number. Premixed circuit components were then pooled into the finished LDR. Without any post‐reaction treatment, all testing procedures could be accomplished within a test tube automatically. As shown in the experimental data, genotypes of the same phenotypic class triggered a similar fluorescence pattern within 2.5 h, which agrees with the expected fluorescence pattern programmed for each class, more importantly, samples tested in different batches could also yield expected fluorescence patterns (Figure 4b). These results suggest the robustness and usefulness of the DNA genetic decoder in real genomic DNA samples. Moreover, we have experimentally demonstrated that the ligase‐based method could transform rare nucleic acid variants with low allele frequencies effectively when combined with blocker displacement amplification (Figure S10a, Supporting Information).^[^ ^26^ ^]^ Rare alleles with even 1% VAF were demonstrated to be effectively enriched through BDA (Figure S10b, Supporting Information), and can trigger accurate multiplicative operations after signal transformation via LDR (Figure S10c, Supporting Information)

### 2.5 Reprogramming DNA Decoding Circuits for Diverse Healthcare Purposes

After experimentally verifying the effectiveness of the DNA‐powered decoding circuit engineered for decoding *CYP2C19* “genetic codes” of human genomic DNA samples, we wondered whether the DNA‐based genetic decoder could be readily reprogrammed for diverse healthcare purposes. Since all components are simple DNA duplexes and highly modular, we expect that programs for more complicated tasks can be reconfigured by simply rewiring or tuning concentrations of circuit modules.

**Figure 5:**
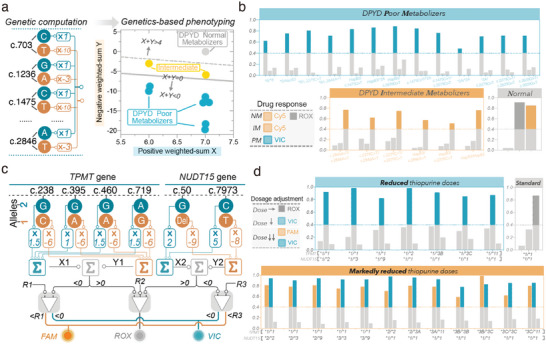
Reprogramming the DNA decoding circuit for precision medicine. a) The DNA decoding circuit for the interpretation of three DYPD phenotypic classes in the weighted‐sum space. Two parallel hyperplanes for separating three types of DYPD metabolizers were shown. Weighted‐sum (*X+Y*) of DPYD normal metabolizers, poor metabolizers, and intermediate metabolizers are “>4”, “<0”, and “>0 but <4”, respectively. b) Experimental validation of fluorescence outputs of the DNA decoding circuit programmed for the interpretation of *DYPD* “genetic codes” using synthetic input patterns. Supposed fluorescence patterns for each phenotypic class were indicated. c) Schematic of a DNA decoding circuit for interpreting the “genetic codes” of *TPMT* and *NUDT15* into recommendations on thiopurines dosages. Detailed “genetic codes” and the supposed weighted‐sum of each *TPMT* and *NUDT15* genotype were listed in Tables S6 and S7, Supporting Information. d) Experimental validation of fluorescence outputs of the DNA decoding circuit programmed for interpreting the “genetic codes” of *TPMT* and *NUDT15* into recommendations on thiopurines dosages. Synthetic input patterns were used for all cases. All data shown were end‐point (2.5 h) relative fluorescence units (R.F.U).

In our initial design, the decoding circuit was programmed to interpret 4 phenotypic classes from various sets of *CYP2C19* “genetic codes.” For other PGx‐genes, phenotypic classes may be annotated in different ways. In the case of *DPYD*, there are three likely phenotypic classes (normal, intermediate, poor), which are classified based on activity scores of *DPYD* genotypes.^[^ ^27^ ^]^ Using the *DPYD* genotype‐phenotype dataset as input, an optimal array of allelic coefficients can be identified by in silico learning. With the fitted coefficient array, three phenotypic classes were observed to be well separated in the weighted‐sum space (**Figure** **5**a). From this, we reprogrammed a decoding circuit to interpret 30 sets of DPYD “genetic codes” into 3 phenotypic classes based on 14 alleles caused by 7 *DPYD* SNPs (Table S6, Supporting Information) and then predicted circuit behaviors using the CRN simulator.^[^ ^28^ ^]^ The simulation can model multiple sets of strand‐displacement reactions and predict circuit behaviors to facilitate circuit optimization.^[^ ^29^ ^]^ The circuit was predicted to perform desired computation for the allele‐specific input set of each genotype and generate the correct fluorescence pattern programmed for the phenotypic class of each genotype (Figure S11, Supporting Information). When synthetic input combinations of *DPYD* genotypes were introduced, the circuit was experimentally observed to output expected fluorescence patterns (Figure 5b).

Furthermore, we expected that the circuit output could shift from the phenotype of drug responses to the dose of a specific medication. Phenotypic variations caused by a single PGx gene may affect the usage of numerous drugs, and likewise, the dose of a single drug is often influenced by several PGx genes. Therefore, we explored the potential to provide dosage recommendations on thiopurines, whose effectiveness and toxicity have been verified to interact with two PGx genes—*TPMT* and *NUDT15*.^[^ ^30^ ^]^ To reprogram a decoding circuit for dose recommendations, we identified coefficient arrays for 8 *TPMT* and 4 *NUDT15* alleles individually using the abovementioned script (Table S7, Supporting Information). The circuit was designed to calculate a weighted‐sum for genotypes of both genes in parallel, compare the weighted‐sum separately when either weighted‐sum is negative (X1+Y1<0 or X2+Y2<0) but compare the weighted‐sum collectively if both weighted‐sum are positive (X1+Y1>0 and X2+Y2>0) (Figure 5c). This is because the normal dose is only applicable to normal metabolizers (X1+Y1>0 and X2+Y2>0) of TPMT and NUDT15, whereas dose reductions are recommended for poor and intermediate metabolizers (X1+Y1>0 or X2+Y2>0) of either TPMT or NUDT15 (Table S8, Supporting Information).^[^ ^30^ ^]^ Circuit simulations predicted correct fluorescent patterns for input combinations of all given genotypes (Figure S12, Supporting Information), and experimental validation using synthetic input combinations of given *DPYD* genotypes demonstrates expected fluorescence patterns programmed for each recommended dosage (Figure 5d). Another two linear models were also successfully trained for interpreting *SLCO1B1* and *UGT1A1* genetic profiles into dose recommendations (Figure S13, Supporting Information), suggesting that the DNA decoding circuit is highly programmable to guide personalized healthcare.

**Figure 6:**
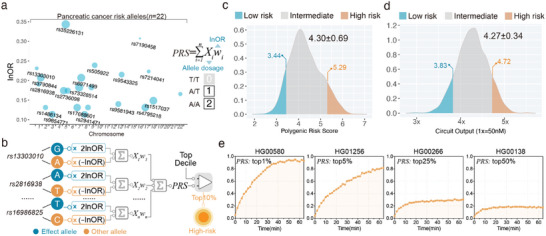
Assessing disease susceptibility based on the molecular calculation of polygenic risk score with the DNA‐powered decoding circuit. a) A set of risk alleles implicated in pancreatic cancer. The polygenic risk score (PRS) for pancreatic cancer (PGS000083) is calculated based on the sum of all risk variants individually multiplied by their effect weight (lnOR). Allelic dosage represents the copy number of the effect allele: “0” for the absence of the allele, “1” for heterozygote, and “2” for homozygote. Dots are sized by allele frequency (the higher, the larger). b) Schematic of a DNA computing circuit programmed for the molecular calculation of the PRS of pancreatic cancer and exporting fluorescent signals for the high‐risk populations (top10%). c) Stratification of the 1000 Genomes Project phase 3 individuals (*n* = 2504) based on theoretical PRS. Polygenic risk categories: low (bottom decile), intermediate (2nd–8th decile), or high (top decile). Mean ± standard deviation and top/bottom decile were indicated. d) Distribution of simulated end‐point (4 h) concentrations of PRS species across the individuals from the 1000 Genomes Project phase 3 (*n* = 2504). Mean ± standard deviation and top/bottom decile were indicated. Polygenic risk categories were defined as above. Simulations were performed at 1× = 50 nm. e) Experimentally characterized circuit behaviors of the DNA‐based genetic decoder programmed for screening high‐risk populations based on PRS level. Synthetic DNA (50 nm for each input strand) was used as the input patterns. Concentrations of multiplicative gates for effect alleles and other alleles were given at 2lnOR*100 nm and lnOR*100 nm. Detailed genotypic data were available in Table S9, Supporting Information.

In addition to guiding drug regimes, another rapidly emerging precision medicine tool is the polygenic risk score (PRS), which estimates a person's genetic risk of developing a particular disease by cumulating the effects of various risk alleles with relatively small individual effects on incidence.^[^ ^31^ ^]^ Though the clinical validity and utility of polygenic risk profiling for risk stratification, screening, and prediction have been evaluated in detail,^[^ ^32^ ^]^ it remains challenging to implement PRS in routine clinical and public health practice,^[^ ^33^ ^]^ which prompted us to explore the utility of DNA decoding circuit in the molecular calculation of PRS and PRS‐informed disease screening, considering its high programmability and modularized circuit layout. We thus abstracted a PGS scoring system for pancreatic cancer from the Polygenic Score Catalog (http://www.pgscatalog.org).^[^ ^34^ ^]^ This PGS scoring system includes 22 risk variants with varying effect sizes (Odds Ratio, OR), and PRS could be calculated as a weighted‐sum score of all effect alleles using their effect sizes from GWAS as weights (**Figure** **6**a). Notably, the allelic dosage was defined by the allele copy number (that is, “1” for a heterozygote and “2” for a homozygote), whereas each allele has a binary state of 0 or 1 in our previously engineered circuit. To achieve a ternary input mode, the multiplicative coefficient of effect alleles was programmed to double its original lnOR while the coefficient of other alleles was set to have a negative lnOR (Figure 6b). In this way, the concentration of weighted species is 2lnOR if the allele is homozygous but reduces to lnOR if the allele is heterozygous, and there are none of positively weighted species in the absence of the allele. Weighted species of each risk locus were then integrated by a summation gate, thereby setting the stage for the molecule calculation of PRS (Figure 6b). Using synthetic input patterns to simulate homozygous/heterozygous allele samples, we experimentally demonstrated that the DNA circuit could implement reliable molecular calculation of risk score for individual risk locus (Figure S14, Supporting Information). We also simulated the circuit behavior with the genotypic dataset of 2504 individuals from the 1000 Genomes Project Phase 3 and statistically compared output concentrations of weighted‐sum species with their supposed PRS(s), which were observed to be normally distributed (mean: 4.30; standard deviation: 0.69) (Figure 6c). Risk stratification based on supposed PRS(s) of the 2504 individuals resulted in three categories: low (bottom decile), intermediate (2nd–8th decile), and high (top decile) (Figure 6c), and we noted that the stoichiometric quantity of the weighted‐sum output was near to the supposed PRS for most test cases and demonstrated a similar distribution with that of supposed PRS (mean: 4.27; standard deviation: 0.34) (Figure 6d). Accordingly, we reasoned that high‐risk individual with top10% PRS could be efficiently screened by fluorescent outputs when a comparator module was incorporated into the circuit (Figure 6b). Using synthetic input patterns of individuals with varying PRS levels (Table S9, Supporting Information), circuit behaviors were expectedly demonstrated to have an ON trajectory in test cases with top1% PRS and top5% PRS, while maintaining an OFF state for test cases with intermediate PRS (Figure 6e), suggesting that the DNA‐powered genetic decoder has great potential to efficiently and straightforwardly implement PRS‐informed individualized disease risk prediction and perform populational risk stratification. Compared with the well‐established Sanger sequencing for genetic testing and interpretation (Table S10, Supporting Information), our DNA‐computation‐based platform shows advantageous features including high multiplexity, automatability, and target expandability, as well as fast turnaround time.

## 3 Discussion

Herein we present a programmable DNA decoding circuit for straightforward genotype‐phenotype interpretation. We have validated the effectiveness of the DNA‐powered genetic decoder in human genomic DNA samples and demonstrated that the intelligent genetic decoder can be readily reconfigured for diverse genetic services, like individualized medication and cancer risk assessment.

To enable parallel molecular computation over a given set of human alleles, a signal‐transforming approach based on thermostable ligase was developed. This approach provides a flexible interface for molecular computing platforms to process biological information by circumventing a series of handing procedures to acquire sequence information of each genetic locus, thereby setting the stage for molecular computation in automated and time‐efficient genetic interpretations. We experimentally demonstrated that this approach could efficiently convert up to five *CYP2C19* alleles to recognizable DNA inputs for the circuit in a single reaction. The striking contrast between the thermodynamic favorability of linked and unlinked probes only allows the linked probe to initiate a computing module. Additionally, increasing ligase and primer concentration, as well as extending the annealing time and thermal cycle number, can improve the ligation efficiency. It should be noted that the ligase‐based approach, though validated in the context of single nucleotide variations, is also amenable to transforming other types of genetic variations, such as insertions and deletions, which collectively constitute an overwhelming proportion of human genetic variations.^[^ ^35^ ^]^

Molecular programming enables the DNA decoding circuit to implement efficient and straightforward genetic interpretation at the molecular level based on an interpretable linear model, wherein the weights of each allele are sized by respective effects on phenotypic outcomes. That is, a risk allele may obtain a negative weight while a protective allele gets a positive weight. Accordingly, the DNA decoding circuit can make a reliable prediction based on the overall genetic effect and can be readily reprogrammed to satisfy a wide variety of smart applications by varying allelic weight array and/or rewiring circuit modules. Given the modularity of the circuit layout, weight adjustment at the molecular level can be flexibly achieved by simply tunning concentrations of arithmetic gates rather than rewiring the interlinked network of the entire circuit. Also, modularized circuit design enables successful molecular computation of polygenic risk scores, which is rapidly evolving in the academic field but rarely incorporated into healthcare practice, and when combined with the thresholding module, the screening of risky individuals was successfully realized. Further, rationally designed threshold modules help to satisfy multi‐categorical classification requirements in clinical practice, rather than report an “either‐or” answer (i.e., YES/NO) about phenotypical classes and medical decisions with diverse alternatives. Notably, the massively parallel computing ability of the DNA decoding circuit renders it highly scalable with the number of input alleles for more sophisticated tasks, while not markedly increasing the turn‐around‐time. Instead, the workload required for conventional genotyping techniques increases remarkably with the number of genetic variations. For large‐scale DNA strand‐displacement circuits, of particular note is to minimize unspecific crosstalk and circuit leakage, which may be effectively achieved by carefully checking the reactive orthogonality of DNA molecules, introducing a clamp domain into output strands, and purifying computing components.

With the high‐density storage and massively parallel computing ability, DNA‐based molecular computation has long been considered a powerful substitute for traditional silicon chips, nevertheless, the most competitive advantage of DNA computation in the healthcare field is its bio‐compatibility, making it a good fit for genetic interpretation at the molecular level. We leveraged this strength and successfully realized the full integration of both genetic detection and data interpretation into a single tube, thereby eliminating additional in silico computation over genetic data acquired by specialized equipment. Though the entire workflow used in this study relies on a PCR machine for pre‐amplification and one‐pot LDR and a microplate reader for the fluorescence readout, however, isothermal amplification methods, for example, recombinase polymerase amplification (RPA), utilize engineered recombinase enzyme instead of thermal denaturing to help primers invade into double‐stranded DNA,^[^ ^36^ ^]^ therefore stand as a promising alternative for the isothermal implementation of pre‐amplification and LDR. While fluorescence spectroscopy is conventionally used for the readout of DNA computing systems, there are many emerging alternatives for point‐of‐care readout,^[^ ^37^ ^]^ for example, a DNA‐based motor can convert computational results into mechanical motions that can be readily visualized by smartphone camera,^[^ ^37b^ ^]^ thereby largely reducing the instrument required for the readout of molecular computing platforms. Also of note is that the turn‐around time for the DNA computing system can be dramatically sped up from hours to minutes by a cyclic freeze/thaw approach.^[^ ^38^ ^]^ These open up the possibility of the molecular computing system for wide‐scale cell‐free scenarios, including DNA methylation‐based in vitro epigenetic diagnostics, noninvasive prenatal screening, and molecular subtyping for precise therapeutic implications. Further, all step‐by‐step procedures can be integrated into a completely automated workflow assisted by the robotic liquid handling system, revealing the supremacy of our DNA computation platform in high accuracy and automation level.^[^ ^39^ ^]^ Given all that, the DNA‐based bio‐computing platform stands as a real‐world demonstration of the utility of molecular computing systems in the intelligent genotype‐phenotype translation of human genomic samples. It can be envisioned to facilitate personalized medication, genetic counseling, and disease risk prediction in primary care and will inspire more powerful and intelligent healthcare applications for improving health outcomes for the general public.

## 4 Experimental Section

### Human Genomic DNA and Synthetic Reference Samples

For the preparation of synthetic DNA samples, a 4.81 kb DNA fragment spanning the partial wild‐type *CYP2C19* gene was chemically synthesized and cloned into the commercial pUC57 vector, resulting in a plasmid referred to as pUC57‐CYP2C19‐WT. The resulting plasmid then served as a template for generating single nucleotide variants via site‐directed mutagenesis, resulting in another three plasmids carrying mutation at different locations: pUC57‐CYP2C19*2, pUC57‐CYP2C19*3, and pUC57‐CYP2C19*17. After sequence verification, these successfully constructed plasmids were respectively transformed into and propagated in the *Escherichia coli* Top10 strain to produce a large number of plasmids used as the DNA template in ligase‐dependent reactions. For the preparation of human genomic DNA samples, 500 µL human peripheral intravenous blood samples were used to extract genomic DNA using a blood genomic DNA extraction kit (Tiangen Biotech, DP348). This study was approved by the Ethics Committee at Renji Hospital, School of Medicine, Shanghai Jiao Tong University (RA‐2021‐103). Informed consent forms were obtained from all human participants recruited in this study. *CYP2C19* genetic polymorphisms of each sample were verified using Sanger sequencing.

### Preamplification and SNP Genotyping of Genomic Samples

The PCR for the preamplification and SNP genotyping of *CYP2C19* with Sanger sequencing was performed using 0.02 units µL^−1^ Q5 polymerase (New England Biolabs, M0491S), 1× Q5 reaction buffer, 200 nm forward and reverse primers (detailed sequences were listed in (Table S2, Supporting Information), and 200 µm dNTPs. The thermal cycling program consisted of 98 °C for 30 s, followed by 30 cycles of 98 °C for 10 s, 65 °C for 30 s, and 72 °C for 20 s.

### Primer Design for Ligase‐Dependent Reaction

Ligase‐dependent reaction (LDR) is used to transform allelic information into corresponding ssDNA inputs for DNA multiplication gates. Each allele has a specific upstream primer which has a particular toehold domain on its 5’ end, and a downstream primer whose 3’ end has been phosphorylated. The upstream allele‐specific primer was expected to be linked with the downstream primer by 9°N ligase. Of note is that the downstream primer is shared by the two alternative alleles at the same locus. To design primer pairs for multiplex alleles, sequences flanking the polymorphic locus of each allele were obtained from NCBI's dbSNP database (https://www.ncbi.nlm.nih.gov/snp/). The primer melting temperature (*T*_m_) of each primer was then estimated by the online reaction temperature calculator (https://ligasecalc.neb.com/#!/ligation). *T*_m_ of all primers was set to 55 °C, resulting in a list of primer candidates. Next, all candidates of linked primers were subjected to a check of secondary structure at 37 °C by NUPACK.^[^ ^40^ ^]^ For linked primers with hairpin structures, intentional mismatches were introduced into the stem domain to make the hairpin structure thermodynamically unfavorable. Note that the base at the 3’ end of all allele‐specific primers should be exactly complementary to the targeted allele. Then, the Tm of each primer candidate with intentionally induced mismatches at the stem domain was then estimated by the reaction temperature calculator (https://ligasecalc.neb.com/#!/ligation). Since a single mismatch can influence Tm dramatically, concentrations of primes with a single mismatch were increased to ensure the hybridization efficiency of the primers to the target template. The incubation temperature of all primer pairs should be within a similar range (50 °C ± 3 °C).

### Ligase‐Dependent Reaction

Ligase‐dependent reaction were performed using 1.6 units µL^−1^ 9°N ligase (New England Biolabs, M0238S), 1 × 9°N ligase buffer, 5 nm plasmid DNA and all necessary primer pairs. Detailed sequences of all primers were specified in Table S2, Supporting Information. The thermal cycling program consisted of 94 °C for 5 min, followed by 30 cycles of 94 °C for 10 s and 50 °C for 90 s. The volume of LDR product used for 100 µL DNA circuit was 20 µL.

### Sequence Design

For all sequences, no more than four A or T and no more than three C were used in a row. Use A, C, and T only for all output strands to reduce secondary structures and undesired interactions; Keep GC content at 30–70% for gate strands. Any two sequences in a sequence pool should not share more than 35% of the domain length.

### Purification of DNA Duplexes

DNA oligonucleotides were chemically synthesized and purified by Sangon Biotech. All unmodified DNA oligonucleotides were purified using ULTRAPAGE, and modified DNA oligonucleotides were purified using HPLC. All oligonucleotides were suspended at 100 µm in 1× TE buffer with 12.5 mm MgCl_2_ and stored at −20 °C. Top and bottom strands of DNA duplexes were pre‐mixed in a 1:1 ratio and annealed at 20 µm in a thermal cycler by heating to 95 °C for 5 min and then ramping down to 25 °C at a rate of 1 °C per 1 min. Purification of DNA duplexes was subsequently performed using 12% native polyacrylamide gel electrophoresis, and then double‐stranded bands were carefully cut out and transferred into 1× TE buffer with 12.5 mm MgCl_2_ to allow free diffusion of DNA out of the gel. Absorbance at 260 nm was determined by NanoDrop One (Thermo Fisher) for quantitation of DNA duplex concentration using extinction coefficients. All purified DNA gates were stored at 4 °C for later use. Detailed sequences of DNA strands used in the circuit were listed in Table S11, Supporting Information.

### Fluorescence Kinetics

Kinetic fluorescence measurements were performed using the Synergy H1 Hybrid Multi‐Mode Reader (BioTek). Unless otherwise specified, all reactions were carried out in 1× TE buffer with 12.5 mm Mg^2+^ and kept at 37 °C throughout the reaction. Excitation/Emission wavelengths were set to 493/522 nm for FAM, 535/575 nm for VIC, 585/615 nm for ROX, and 643/671 nm for Cy5. The master mixture containing all circuit components, except input strand(s) or LDR products, was initially added to the 96‐well all‐black plate to record the background fluorescence, followed by the addition of input strand(s) or LDR products. Fluorescence signals were thereafter collected every 2 min for each well.

### Statistical Analysis

Raw fluorescence signals were normalized into the standard concentration of respective output strands by performing linear fitting based on a calibration curve constructed using the steady‐state fluorescence levels of reaction mixtures with a series of reporter initiator strands of predetermined concentrations as input strand(s). For normalization of raw fluorescence intensities of a given fluorophore into relative fluorescence unit (R.F.U), the baseline fluorescence obtained from the initial measurement of the negative control (no input strands) was treated as the minimum (relative fluorescence unit = 0), while the highest fluorescence level in each set of parallel kinetic fluorescence experiments or fluorescence from the standard reporter initiating strands was treated as the maximum (relative fluorescence unit = 1). Statistical analysis of PRS distribution was performed using RStudio (2022.02.0).

## Conflict of Interest

The authors declare no conflict of interest.

## Supporting information

Supporting Information

## Data Availability

The data that support the findings of this study are available from the corresponding author upon reasonable request.
