# Supplementary material for: Unlocking Genetic Profiles with a Programmable DNA‐Powered Decoding Circuit
Source: Adv Sci (Weinh). 2023 Apr 28;10(20):2206343. doi: 10.1002/advs.202206343 (PMC10369254; doi:10.1002/advs.202206343)
Supplement: Supplementary file 1 — Supporting Information [file ADVS-10-2206343-s001.pdf]

ADVANCED SCIENCE
Open Access

Supporting Information

for *Adv. Sci.*, DOI 10.1002/advs.202206343

Unlocking Genetic Profiles with a Programmable DNA-Powered Decoding Circuit

Junlan Liu, Chao Zhang, Jinxing Song, Qing Zhang, Rongjun Zhang, Mingzhi Zhang, Da Han and Weihong Tan**

Supplementary Information for

Unlocking genetic profiles with a programmable DNA-powered decoding circuit

Junlan Liu[a], Chao Zhang[a], Jinxing Song[a], Qing Zhang[a], Rongjun Zhang[a], Mingzhi Zhang[a], Da Han[a,b,*] and Weihong Tan[a,b,c,*]

[a]Institute of Molecular Medicine (IMM), Renji Hospital, Shanghai Jiao Tong University School of Medicine, and College of Chemistry and Chemical Engineering, Shanghai Jiao Tong University, Shanghai 200240, China
[b]The Key Laboratory of Zhejiang Province for Aptamers and Theranostics, Zhejiang Cancer Hospital, Hangzhou Institute of Medicine (HIM), Chinese Academy of Sciences, Hangzhou, Zhejiang 310022, China
[c]Molecular Science and Biomedicine Laboratory (MBL), State Key Laboratory of Chemo/Biosensing and Chemometrics, College of Chemistry and Chemical Engineering, College of Biology, Aptamer Engineering Center of Hunan Province, Hunan University, Changsha, Hunan 410082, China

*Weihong Tan Email: tan@hnu.edu.cn
*Da Han Email: dahan@sjtu.edu.cn

Supplementary Note 1. The MATLAB script for searching fitted coefficient arrays

```
[coef,class] = xlsread('inputfilenamehere.xlsx');
[r,c] = size(class);
A = zeros(r-1,c-1);
PM = [];
IM = [];
EM = [];
UM = [];
for i = 2:r
    if(strcmp(class(i,1),'PM'))
        A(i-1,:) = coef(i-1,1:c-1);
        PM(end+1,:) = coef(i-1,1:c-1);
    end
    if(strcmp(class(i,1),'IM'))
        A(i-1,:) = coef(i-1,1:c-1);
        IM(end+1,:) = coef(i-1,1:c-1);
    end
    if(strcmp(class(i,1),'EM'))
        A(i-1,:) = -coef(i-1,1:c-1);
        EM(end+1,:) = coef(i-1,1:c-1);
    end
    if(strcmp(class(i,1),'UM'))
        A(i-1,:) = -coef(i-1,1:c-1);
        UM(end+1,:) = coef(i-1,1:c-1);
    end
end
%  um > em & im > pm
 [rowOfEM,colOfEM] = size(EM);
 [rowOfUM,colOfUM] = size(UM);
 [rowOfPM,colOfPM] = size(PM);
 [rowOfIM,colOfIM] = size(IM);
 for iEM = 1:rowOfEM
     for jUM = 1:rowOfUM
         tmp = EM(iEM,:) - UM(jUM,:);
         A(end+1,:) = tmp;
     end
 end
 for iPM = 1:rowOfPM
     for jIM = 1:rowOfIM
         tmp = PM(iPM,:) - IM(jIM,:);
         A(end+1,:) = tmp;
     end
 end
[rr,cc] = size(A);
result = [];
for var1 = -10:-1
   for var2 = 0:10
       for var3 = -10:-1
            for var4 = 0:10
                for var5 = 0:10
                     for var6 = 0:10
```

```
                         x = [var1, var2, var3, var4, var5, var6];
                            for k = 1:rr

                                sumOfRowA = sum(A.*x,2);
                                 if(sumOfRowA(k,1) >= 0)
                                        break;
                                 end
                                 if(k == rr)
                                      result(end+1,:) = x(1,:);
                                 end
                            end
                       end
                 end
             end
         end
   end
end
xlswrite('outputfilenamehere.xlsx',result);
```

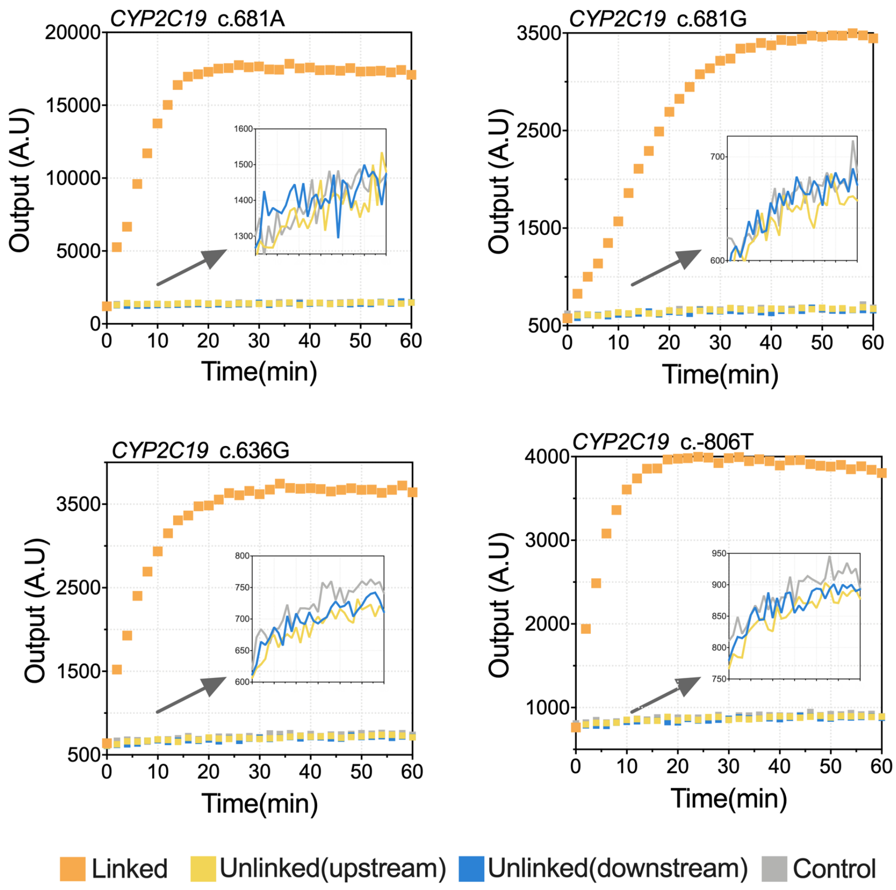

Figure S1 Experimental verification of the ability of linked (10nM synthetic DNA) and inability of unlinked primer (100nM synthetic DNA) to displace corresponding output strand from the multiplication gate designed for indicated *CYP2C19* allele. Kinetic fluorescence experiments were performed at 37℃ in TE buffer with 12.5 mM $MgCl_2$.

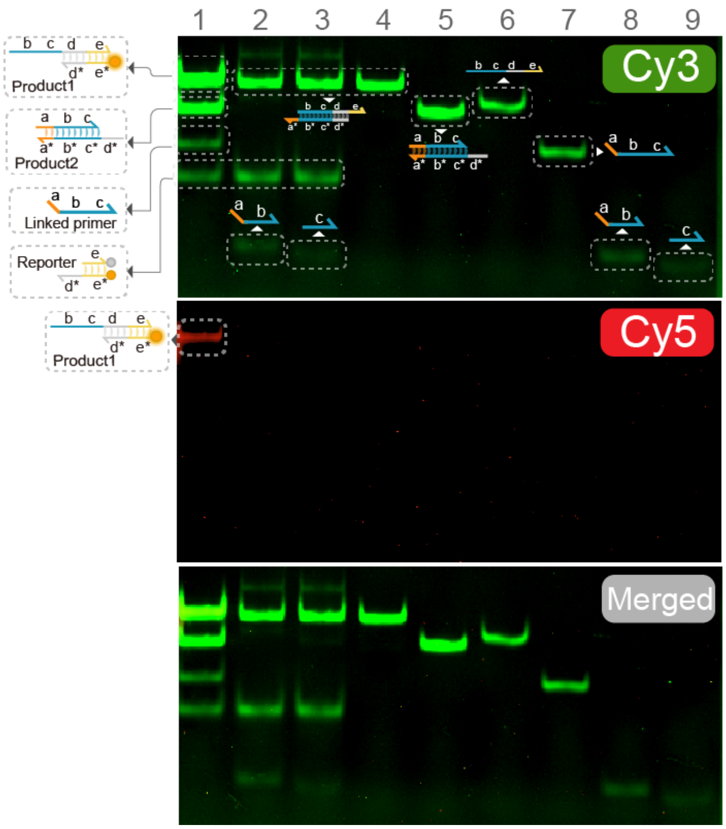

Figure S2. A native PAGE gel showing products of reactions between 100nM DNA multiplicative gate and 200nM linked primers (Lane1), 200nM upstream (Lane2) or 200nM downstream unlinked primers (Lane3). Other reactants include 200nM ROX-labeled reporter duplexes and 12.5mM $MgCl_2$. The reactions were performed at 37°C for 20 minutes. Components of Lane 4 to lane 9 are as follows: 100nM purified DNA gate species (Lane4), 100nM purified duplexes between linked primer and bottom gate strand (Lane5), 200nM output (Lane6), 200nM linked primer (Lane7), 200nM upstream unlinked primer (Lane8), 200nM downstream unlinked primer (Lane9). All given concentrations are based on their final concentrations. Filters used for fluorescent gel imaging were indicated.

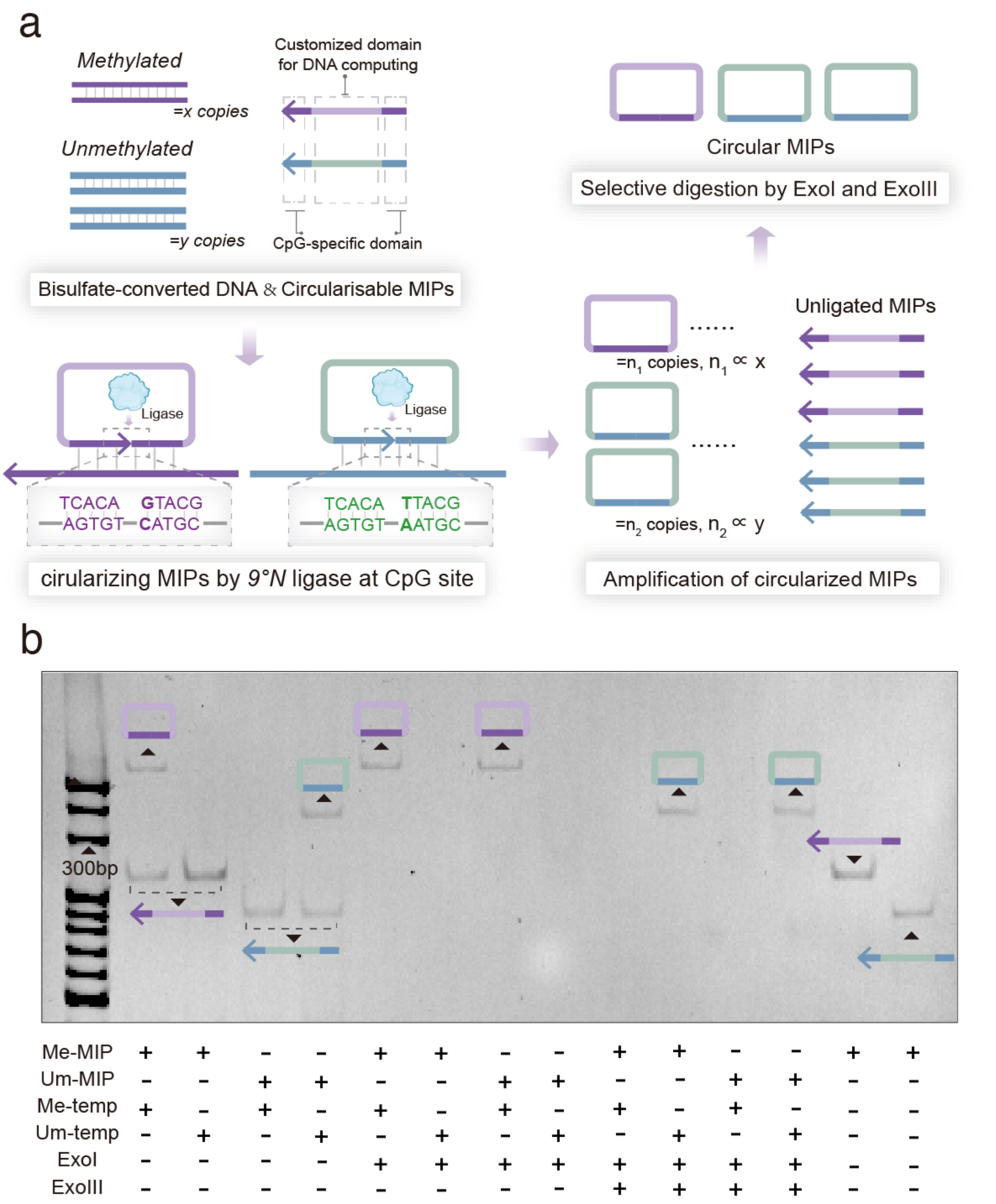

Figure S3. The ligation-based method in combination with molecular inversion probes (MIPs) can transform DNA methylation information into unique circular ssDNA inputs. **a)** Schematic illustration of the amplification and transformation steps for DNA methylation. **b)** Native PAGE analysis of circular ssDNA products amplified and transformed from indicated reaction components and exonuclease treatment. MIP: molecular inversion probes; Me-temp: synthetic methylated template. Um-temp: synthetic unmethylated template.

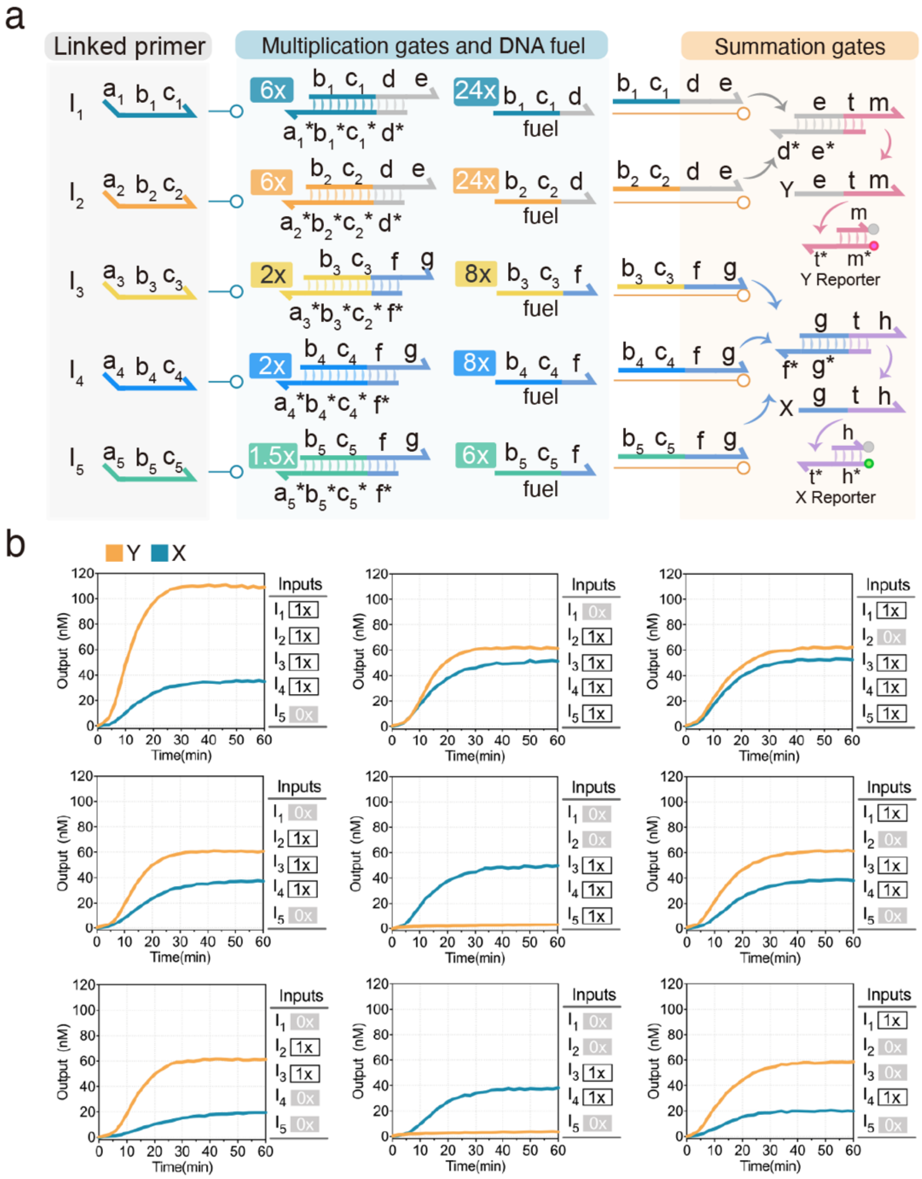

Figure S4 Parallel arithmetic operations over multiple allele-specific linked primers using DNA molecules. **a)** Schematic illustration of multiplication and summation that utilize three types of DNA molecules: (1) Multiplication gates whose concentrations equal multiplicative coefficient, (2) Excessive DNA fuel strands for reuse of inputs strands over multiple catalytic cycles, (3) Summation gates for integration of weighted species. **b)** Fluorescence kinetics of multiplication and summation with indicated combinations of input strands. All experiments were performed at 37°C in TE buffer with 12.5 mM $MgCl_2$. 1x=10nM for all cases.

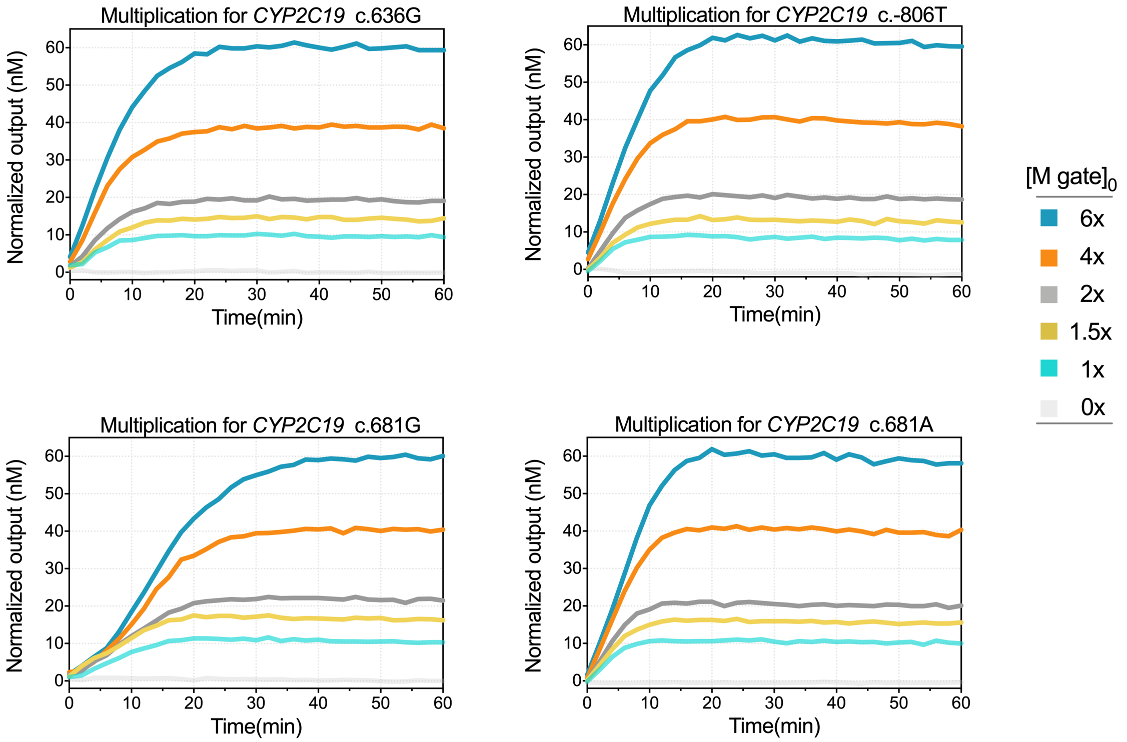

Figure S5 Fluorescence kinetics of four multiplication gates designed for indicated *CYP2C19* alleles. Input strands corresponding to each multiplication gate was given at 10nM, and the initial concentrations (multiplicative coefficients) of multiplication gates were denoted (1x=10nM). All experiments were performed at 37°C.

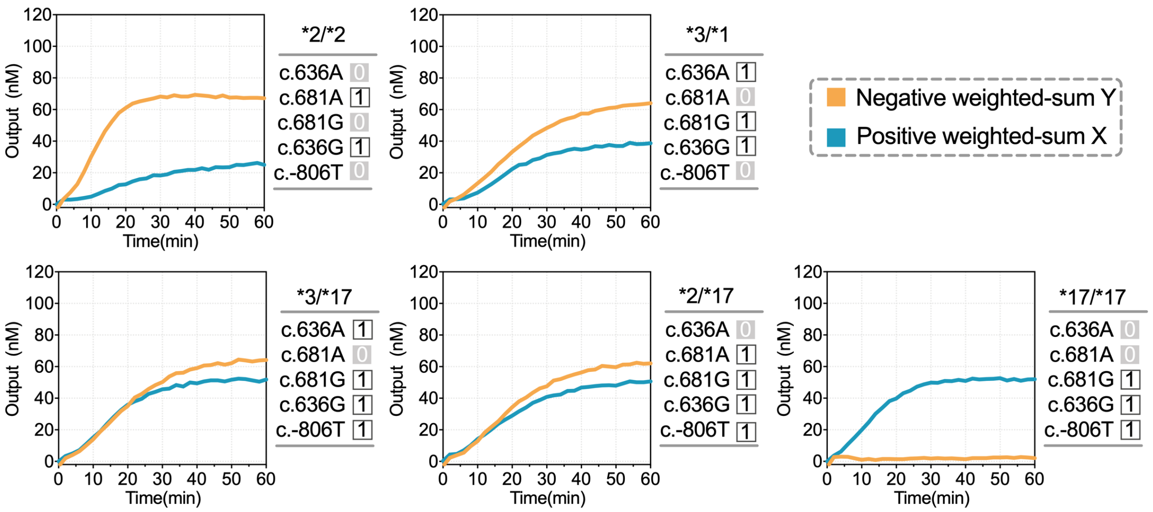

Figure S6 Parallel computation of positive and negative weighted-sum for five *CYP2C19* genotypes (indicated by star alleles). Inputs were 20μL LDR product using premixed plasmids carrying sequences corresponding to indicated diplotypes as template.

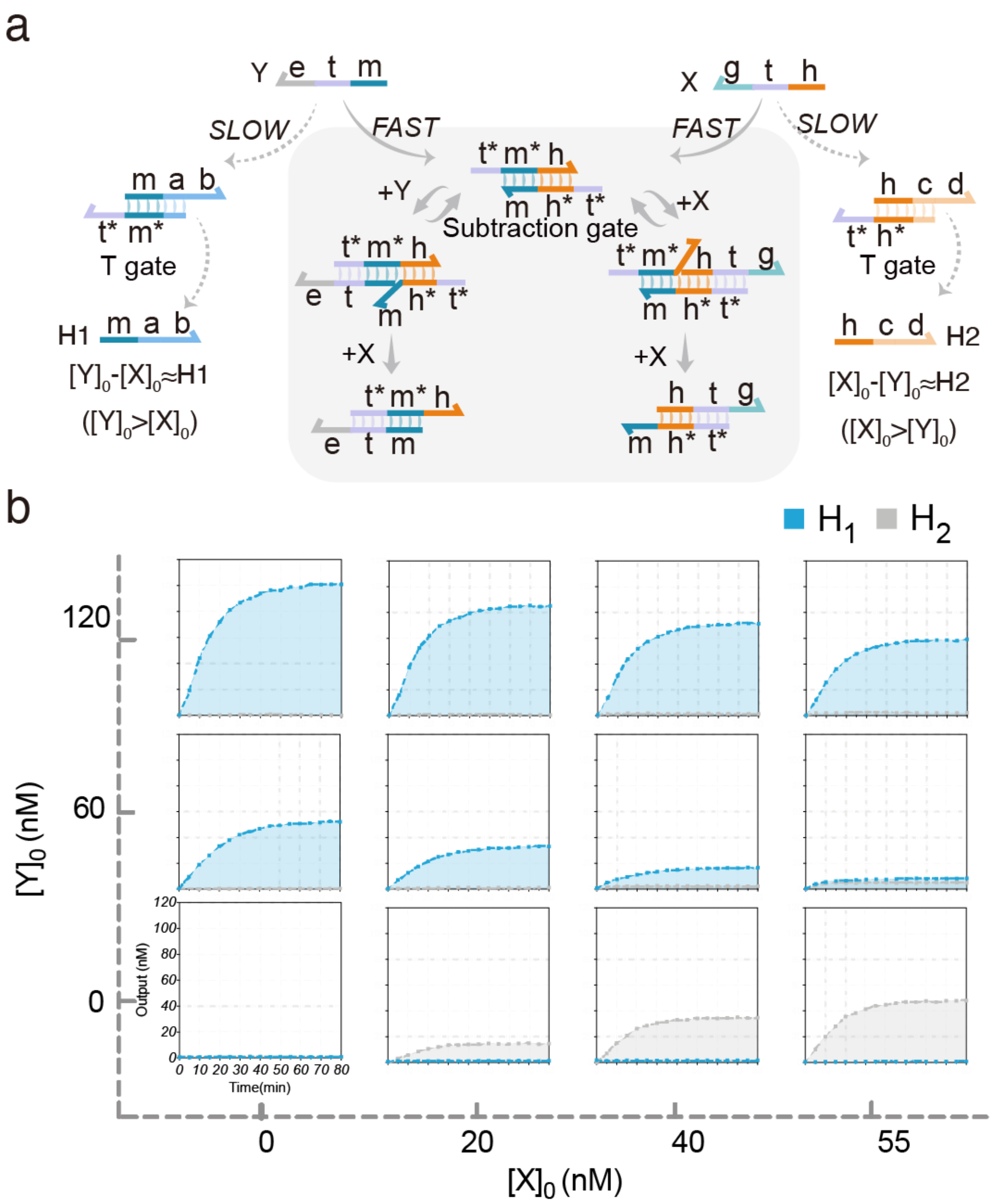

Figure S7 Subtractive operations for computing weighted-sum using DNA molecules. **a)** Abstract diagram of subtraction. Positively weighted-sum species X and negatively weighted-sum species Y cooperatively hybridize with a DNA subtraction gate and whichever in excess can then stoichiometrically displace an equal amount of H probe from T gate. **b)** Experimental validation of subtraction using serial combinations of X and Y at indicated initial concentrations. The x axis denotes time (0–90min) and y axis denotes normalized concentration of H probe (0-120nM).

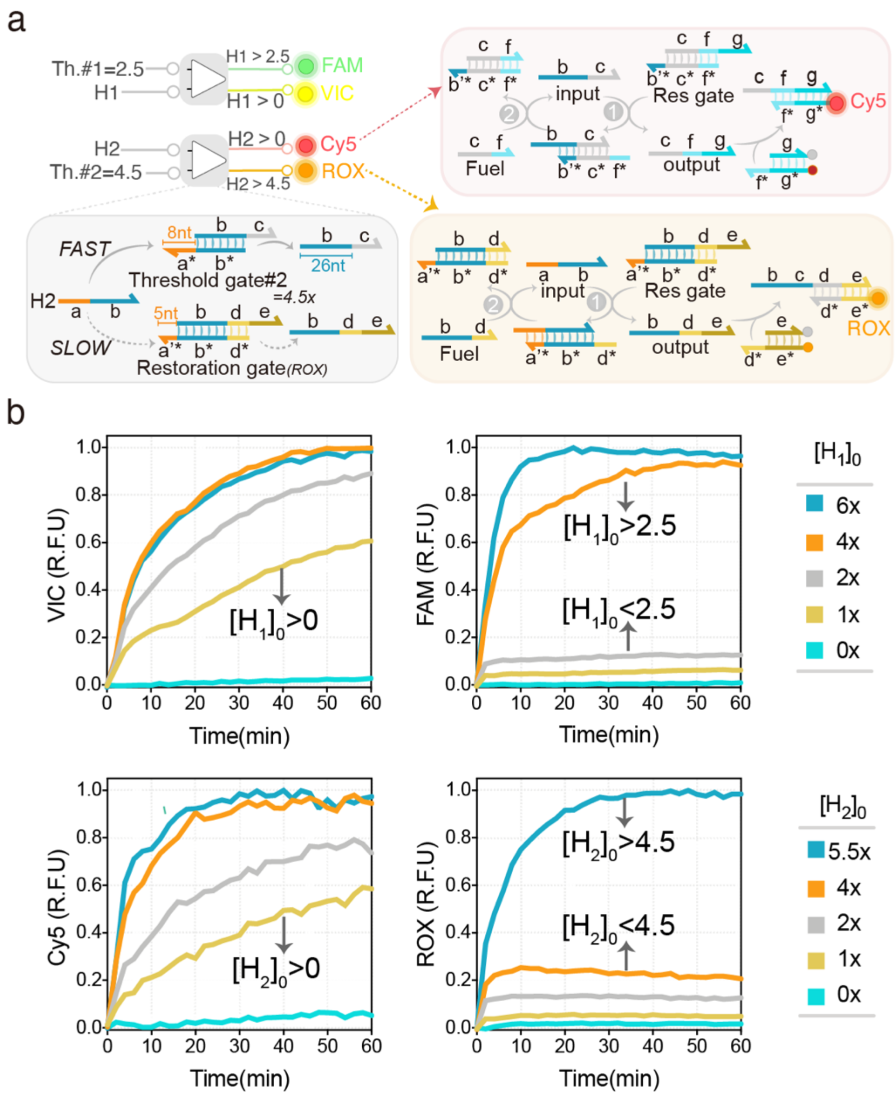

Figure S8 Numeric comparison of weighted-sum. **a)** Schematic illustration of two comparators for signifying distinct numerical intervals of weighted sum using four distinct fluorescence reporters. Each comparator is composed of a threshold gate and two signal restoration gates. Threshold gate #1 and #2 were programmed to accept weighted-sum species H1 and H2 as input respectively. Restoration gates of VIC and Cy5 were programmed to accept the top strand displaced from threshold gate #1 and #2, respectively. **b)** Experimental characterization of thresholding effectiveness using varying initial concentrations of H1 and H2. The concentrations of threshold gate #1 and #2 were fixed at 2.5x and 4.5x, respectively. 1x=10nM for all cases. All experiments were performed at 37℃.

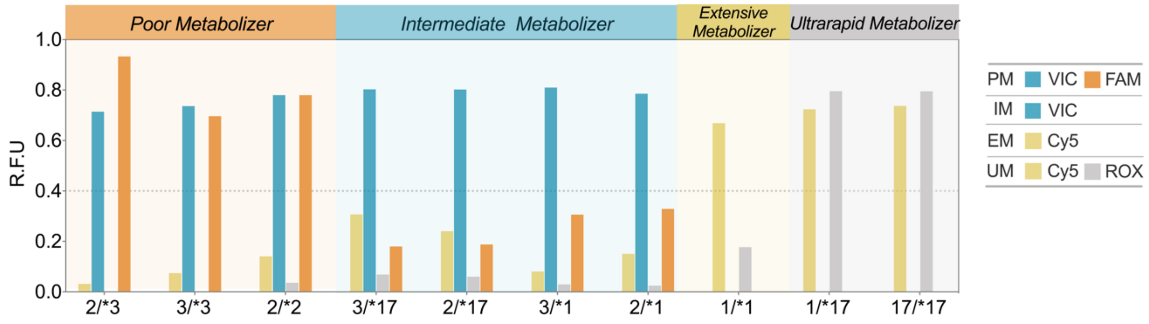

Figure S9 Examining the classification power of the DNA decoding circuit with synthetic inputs. The circuit was programmed to decode *CYP2C19* 'Genetic code'. The inputs were synthetic linked primers pooled according to the possible LDR products (linked primers) of indicated *CYP2C19* genotype. Data shown are end-point (2.5 hours) relative fluorescence units (R.F.U) normalized by fluorescence from negative control (R.F.U=0) and the standard initiator strand of each fluorescent reporter (R.F.U=1).

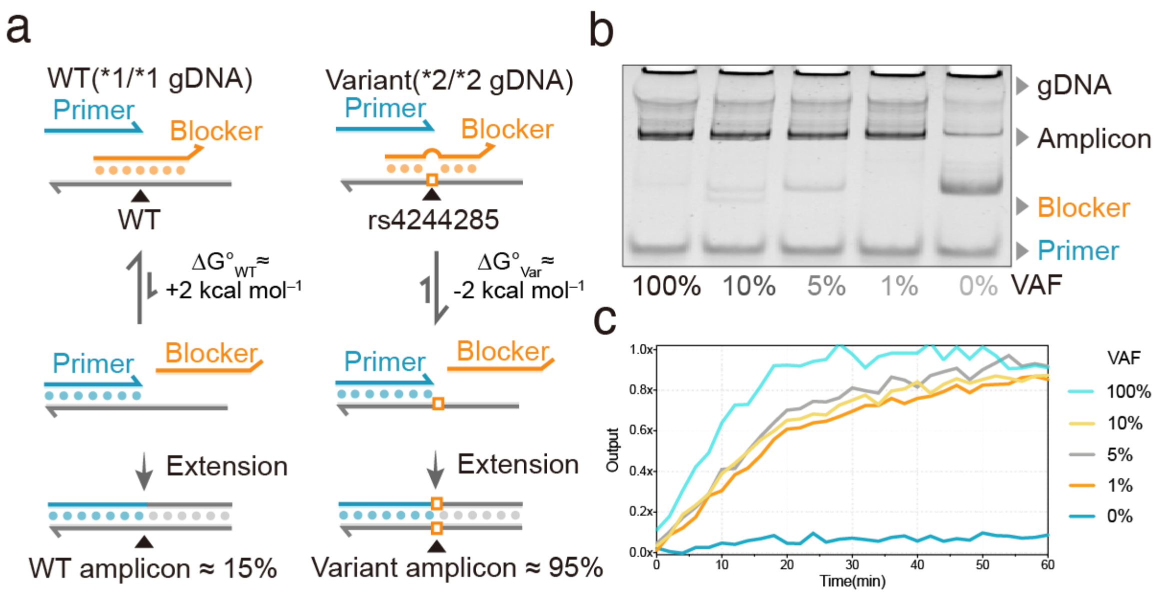

Figure S10 Improved sensitivity of the ligase-based method with blocker displacement amplification (BDA). **a)** Schematic of rare allele enrichment by BDA. The blocker is designed to be perfectly complementary to a known *CYP2C19* WT (*1/*1) template sequence; The 3' end of the primer sequence is identical to the 5' end of the blocker sequence; primer and blocker hybridization to the same template molecule is mutually exclusive while a single-nucleotide variation would cause the ΔG° of the primer displacing the blocker on the variant template (*CYP2C19* *2/*2) to drop off, resulting in a difference in per-cycle amplification yield to realize 1,000-fold enrichment of rare allele. **b)** Polyacrylamide gel electrophoresis (PAGE) analysis of BDA amplicons after 40 rounds of PCR. Variant allele fractions (VAFs) of each sample were indicated. **c)** Fluorescence kinetics of multiplicative operations (weight=1x) with gDNA samples with different VAFs, which were prepared by mixing the WT (1/*1) gDNA and variant (*2/*2) gDNA samples at different ratios and each sample consisted of 200 ng gDNA in total, and was subjected to BDA enrichment before LDR. 1x=20nM for all cases.

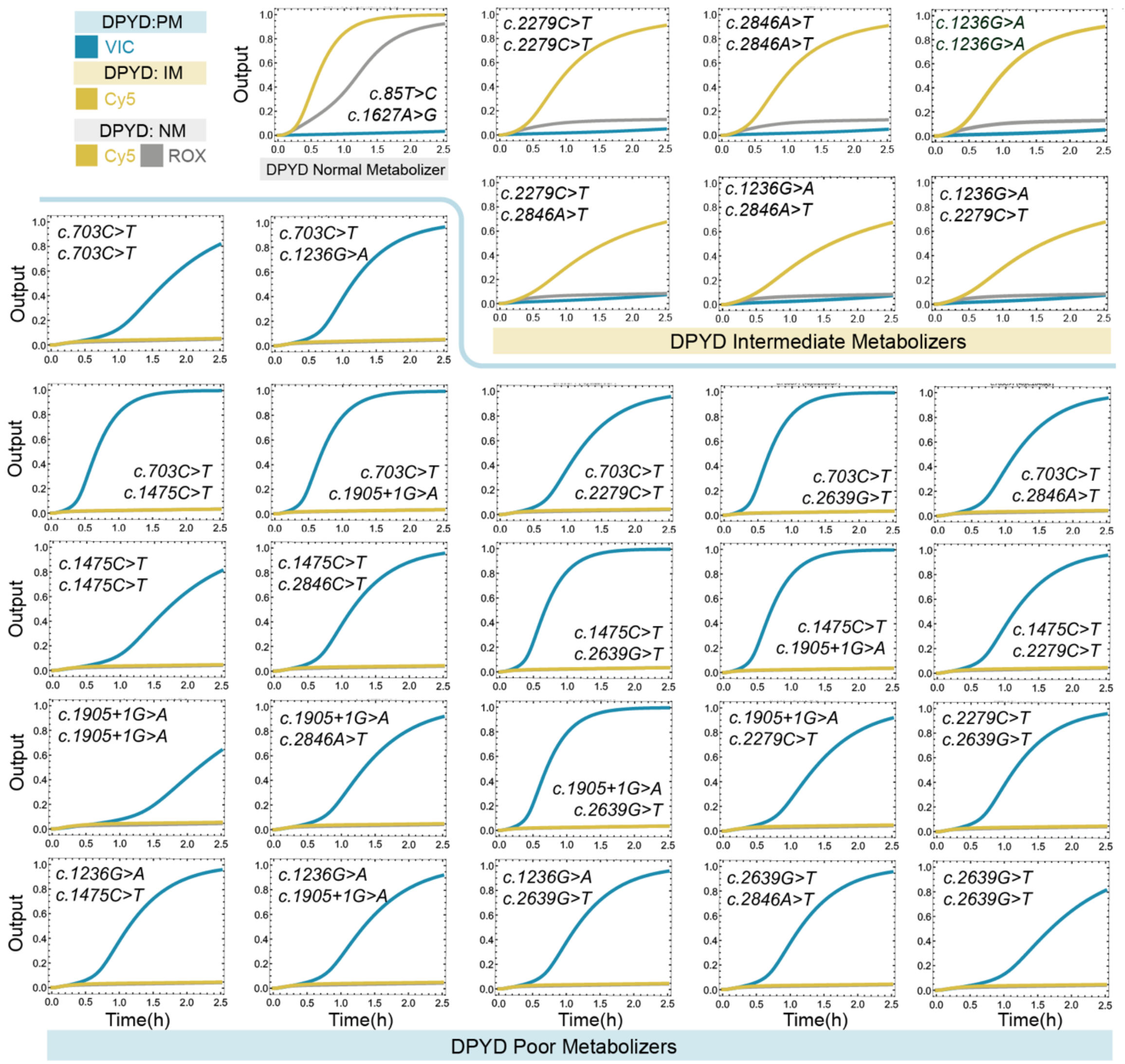

Figure S11 Simulations of the decoding circuit programmed for interpreting *DPYD* 'Genetic code'. Kinetics parameters used for all simulations were fitted from the fluorescence kinetics data of the experimentally characterized *CYP2C19* decoding circuit. The fluorescence pattern preprogrammed for each phenotypic class is indicated. PM: DPYD poor metabolizers; IM: DPYD intermediate metabolizers; NM: DPYD normal metabolizers. Simulations were performed at 1x=10 nM using the CRN simulator (https://users.ece.utexas.edu/~soloveichik/crnsimulator.html).

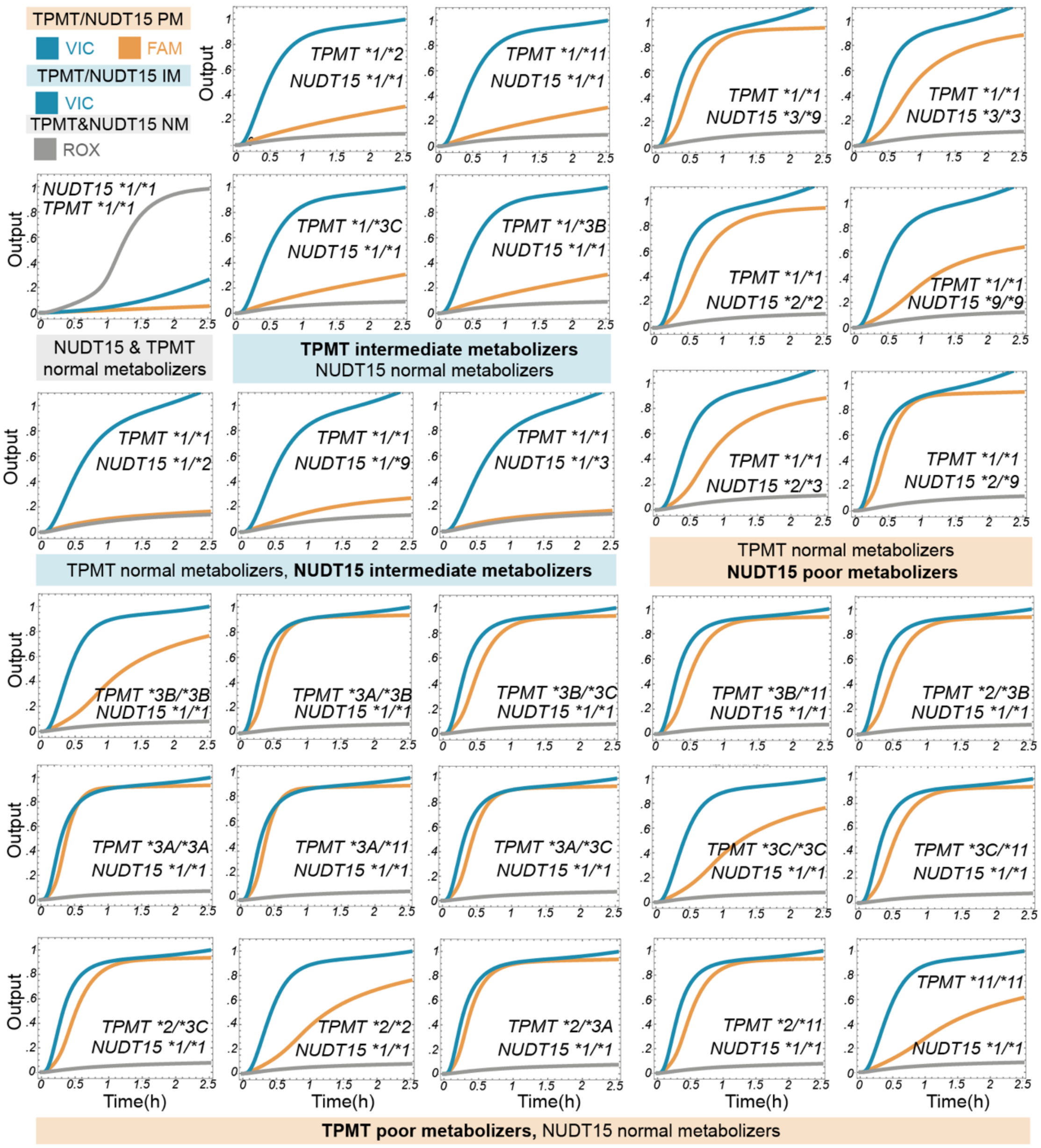

Figure S12 Simulations of the decoding circuit programmed for interpreting *TPMT* and *NUDT15* 'Genetic code' into recommended starting doses of thiopurines. Kinetics parameters used for all simulations were fitted from the fluorescence kinetics data of the experimentally characterized *CYP2C19* decoding circuit. The fluorescence pattern preprogrammed for each phenotypic class is indicated. Markedly reduced dosage is recommended for PM (TPMT or NUDT15 poor metabolizers); reduced dosage is recommended for IM (TPMT or NUDT15intermediate metabolizers); Normal dosage is recommended for NM (TPMT and NUDT15 normal metabolizers). Simulations were performed at 1x=10 nM using the CRN simulator.

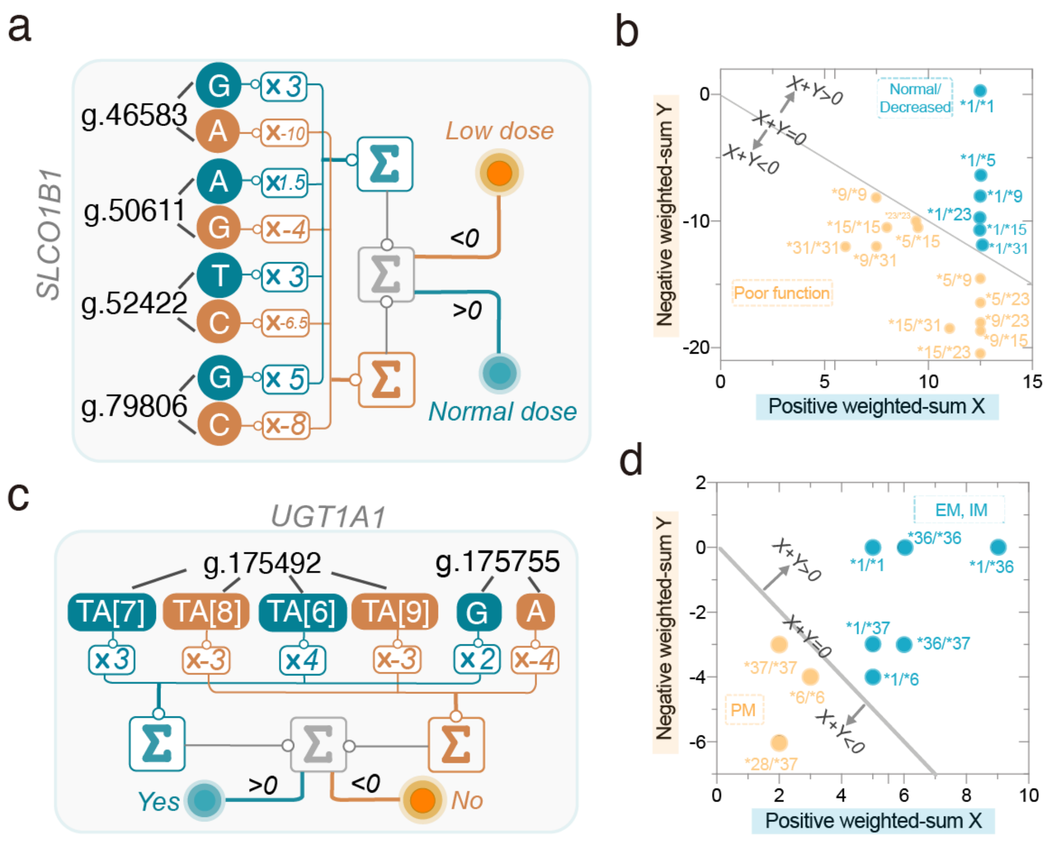

Figure S13 Two linear models for decoding *SLCO1B1* and *UGT1A1* genetic profiles into dose recommendation. **a)** schematic of a circuit designed for decoding *SLCO1B1* alleles into recommendations on pravastatin doses. **b)** a weighed-sum space including different kinds of *SLCO1B1* genotypes. **c)** schematic of a circuit designed for decoding *UGT1A1* into decisions on whether it should use atazanavir (YES) or not (No). **d)** a weighed-sum space including different *UGT1A1* genotypes. PM: poor metabolizers; IM: intermediate metabolizers; EM: extensive metabolizers.

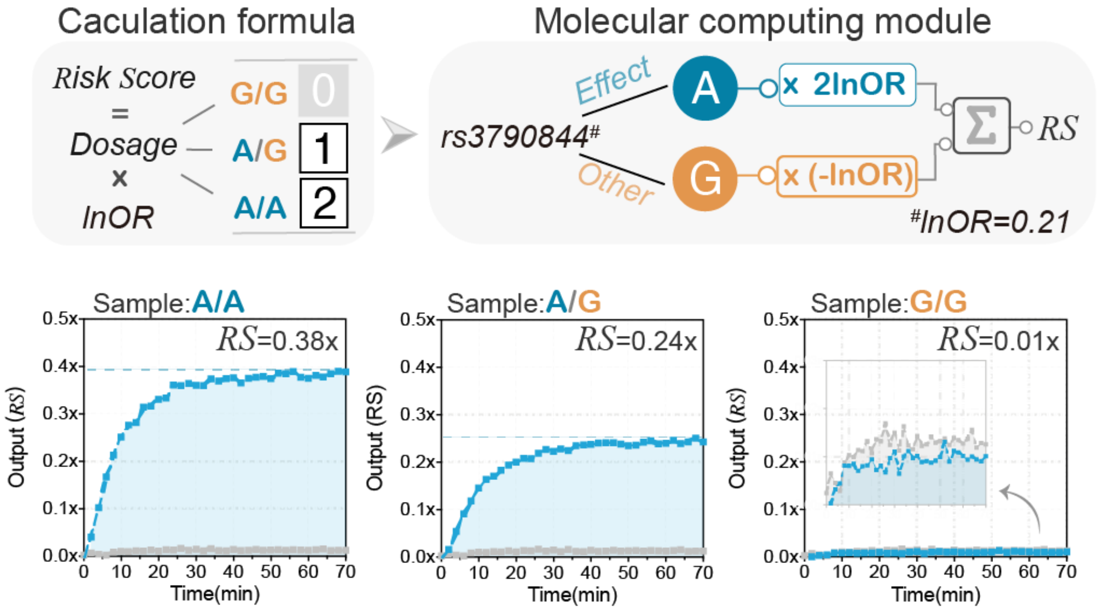

Figure S14 Experimental characterization of a DNA computing module for the molecular calculation of single-locus risk score (RS). The effect size (lnOR) of the indicated genetic variation (rs3790844) is 0.21. Synthetic DNA oligos were used as the input pattern for all test cases (50nM for each input strand). All experiments were performed at 37℃ in TE buffer with 12.5 mM $MgCl_2$. 1x=100nM for all cases.

Table S1. Decoding ‘genetic codes’ written by a battery of *CYP2C19* alleles for interpretation of drug responses

Drug response	Genotype	Effect allele [#1]						Weighted Sum [#2]
		rs4244285		rs4986893		rs12248560		
		c.681A	c.681G	c.636A	c.636G	c.-806T	c.-806C	
Poor Metabolizers	*2/*3	1	1	1	1	0	1	-8
	*3/*3	0	1	1	0	0	1	-4
	*2/*2	1	0	0	1	0	1	-4
Intermediate Metabolizers	*2/*1	1	1	0	1	0	1	-2
	*2/*17	1	1	0	1	1	1	-0.5
	*3/*1	0	1	1	1	0	1	-2
	*3/*17	0	1	1	1	1	1	-0.5
Extensive Metabolizers	*1/*1	0	1	0	1	0	1	4
Ultrarapid Metabolizers	*1/*17	0	1	0	1	1	1	5.5
	*17/*17	0	1	0	1	1	0	5.5

#1: Six SNP features are selected from *CYP2C19* genetic polymorphisms, and according to HGVS nomenclature standard, all features given are described in relation to the coding DNA reference sequence NM_000769.4, with a few modifications wherein the polymorphic position is still denoted by numbering (position in front of the protein coding sequence gets a minus sign), but the letter (nucleotide) denotes DNA code on corresponding loci, rather than indicating a substitution (e.g., ‘c.681A’ indicates the A nucleotide at position c. 681). For each genotype, the logic value of each SNP feature gets ‘1’ as long as either of the two alleles carries the indicated SNP, or otherwise gets ‘0’.
#2: Weights of -6, 2, -6, 2, 1.5, 0 were assigned to ‘c.681A’, ‘c.681G’, ‘c.636A’, ‘c.636G, ‘c.-806T’ and ‘c.-806C’, respectively.

Table S2. Primer sequences used in this study

Purpose	Primer	Sequence (5' to 3')
Ligase-dependent reaction for *CYP2C19* gene	c.681A-specific primer	ACCATACCCATTGATTATTTCCCA
	c.681G-specific primer	CACCACTATTGATTATTTCCCG
	c.681A/G-common primer	(5'-P) GGAACACATAACAAATTACTTAAAA
	c.636A-specific primer	TACCCTCTATCACGATTGTAAGCACTCCCCTGA
	c.636G-specific primer	CACTCCTTATCACGATTGTAAGCACTCCCCTGG
	c.636A/G-common primer	(5'-P) ATCCATGTAAAGCCAAGTTTTTT
	c.-806T-specific primer	ACCTCATCGCATTATTTCTTACATCAGAGATA
	c.-806C/T-common primer	(5'-P) CTTTCAGAACAGAACACACAAATTT
CYP2C19 genotyping with Sanger sequencing	*2-F	AGAGCTTGGCATATTGTATCTATACCTTTAT
	*2-R	CTCCTTGACCTGTTAAACATCCG
	*3-F	AAATTGTTTCCAATCATTTAGCTTCACCC
	*3-R	GCCTGATCTATATTGGGATATTCATTTCCTG
	*17-F	TCTGGGGCTGTTTTCCTTAGATAAAT
	*17-R	CCTAAAAAAACACGTGAAGGCAGG
BDA enrichment for rs4244285	Forward primer	TAATTTTCCCACTATCATTGATTATTTCCC
	Blocker	GATTATTTCCCGGGAACCCATAACAAATTACAATT

Table S3. Approximate concentrations of ligated products after 30 thermal cycles of LDR using indicated primer pairs

	LDR primer pairs for *CYP219* alleles				
	c.681G	c.681A	c.636G	c.636A	c.806T
Band intensity (linked)	2.512	2.158	0.743	0.284	1.107
Band intensity (unlinked)	1.377	2.688	5.080	4.127	3.109
Intensity Ratio (linked/unlinked)	1.82	0.80	0.15	0.07	0.36
Linked primer (nM)	96.9	89.1	89.3	64.4	52.5
Nonspecific ligation events, %	2.1	0.03	0.09	0.5	0.01
Ligation efficiency, %	64.6	59.4	59.5	42.9	35.0

NOTE: Ligation efficiency is the ratio of the actual concentration of linked primer to its supposed concentration (template concentration multiplied by the number of thermal cycles). The template concentration (synthetic plasmids) is 5nM for all test cases. The percent of nonspecific ligation events is the ratio of the actual concentration of nonspecifically linked primers in the context of untargeted template (carrying a single-nucleotide variation) to initial primer concentration. This parameter is used to evaluate the selectivity of LDR primers against point mutations. PAGE image was analyzed using ImageJ [1].

Table S4. Preparation of synthetic plasmid-based genotypic samples for ligase-dependent reactions (The final concentration of 1x is 5nM)

CYP2C19 genotype	Recombinant plasmids			
	pUC57-*1	pUC57-*2	pUC57-*3	pUC57-*17
	c.-806C<br>*c.636G*<br>*c.681G*	*c.-806C*<br>*c.636G*<br>*c.681**A***	*c.-806C*<br>*c.636**A***<br>*c.681G*	*c.-806**T***<br>*c.636G*<br>*c.681G*
*2/*3	0x	1x	1x	0x
*3/*3	0x	0x	1x	0x
*2/*2	0x	1x	0x	0x
*2/*1	1x	1x	0x	0x
*2/*17	0x	1x	0x	1x
*3/*1	1x	0x	1x	0x
*3/*17	0x	0x	1x	1x
*1/*1	1x	0x	0x	0x
*1/*17	1x	0x	0x	1x
*17/*17	0x	0x	0x	1x

Table S5. Sanger sequencing results of 30 clinical human genomic DNA samples

No.	c.681G>A	c.636G>A	c.-806C>T	Haplotype	Phenotype
1	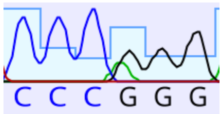	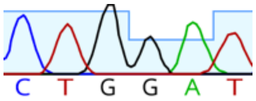	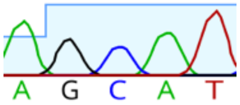	*2/*1	IM
2	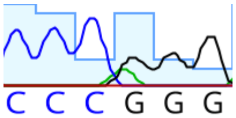	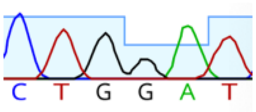	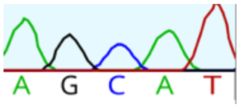	*2/*1	IM
3	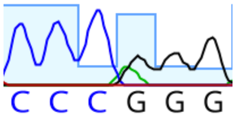	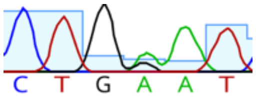	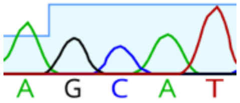	*2/*3	PM
4	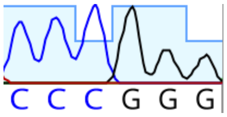	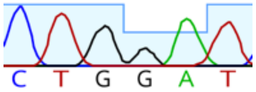	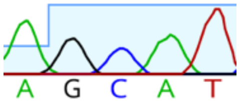	*1/*1	EM
5	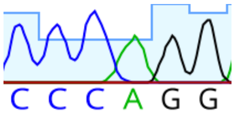	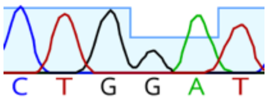	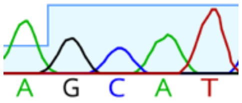	*2/*2	PM
6	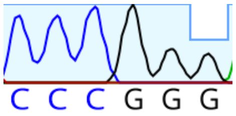	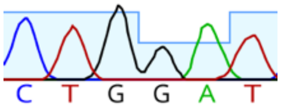	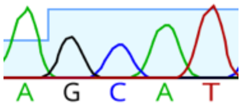	*1/*1	EM
7	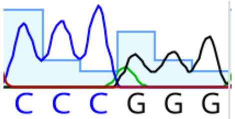	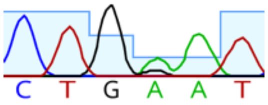	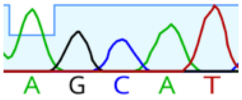	*2/*3	PM
8	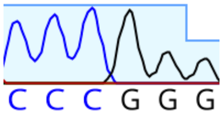	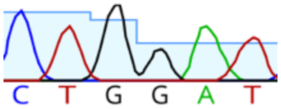	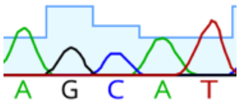	*1/*1	EM
9	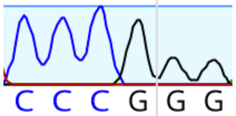	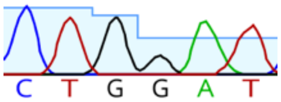	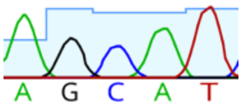	*1/*1	EM
10	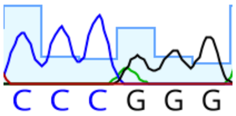	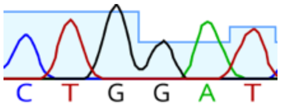	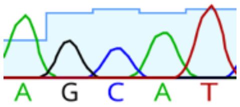	*2/*1	IM

11	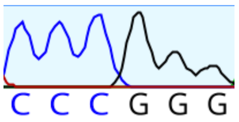	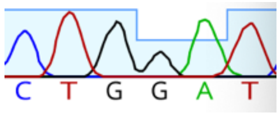	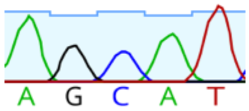	*1/*1	EM
12	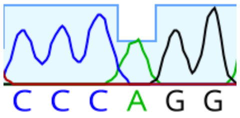	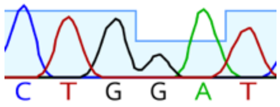	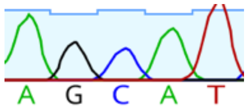	*2/*2	PM
13	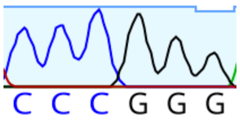	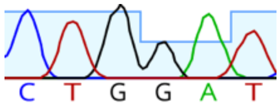	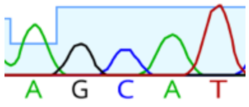	*1/*1	EM
14	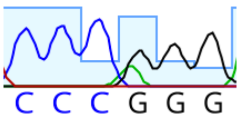	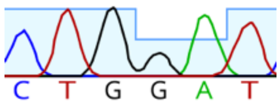	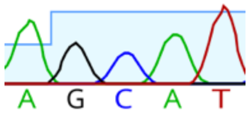	*2/*1	IM
15	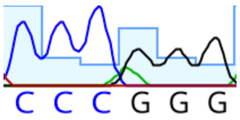	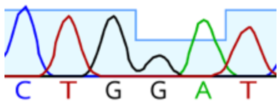	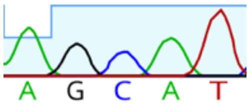	*2/*1	IM
16	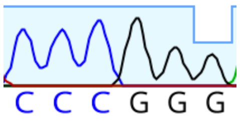	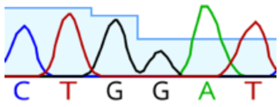	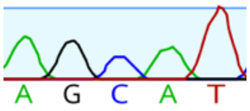	*1/*1	EM
17	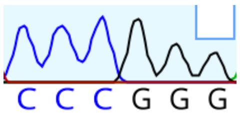	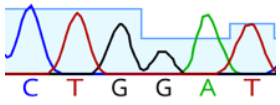	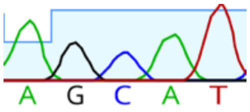	*1/*1	EM
18	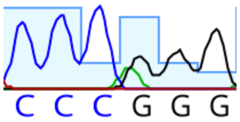	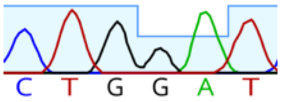	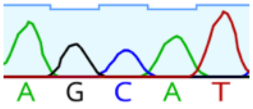	*2/*1	IM
19	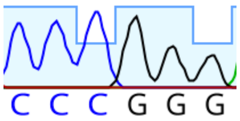	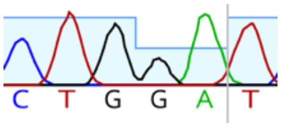	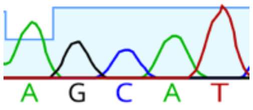	*1/*1	EM
20	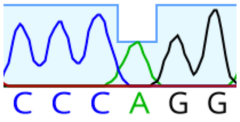	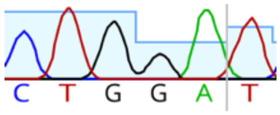	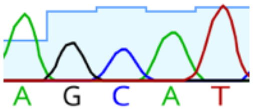	*2/*2	PM
21	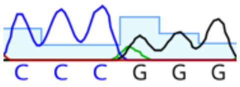	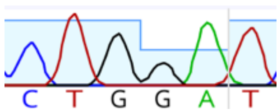	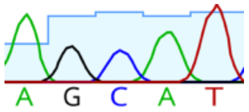	*2/*1	IM

22	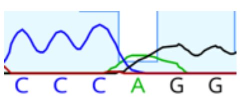	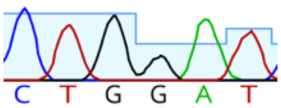	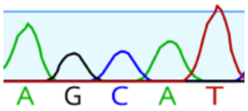	*2/*1	IM
23	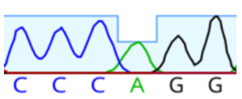	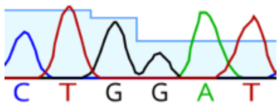	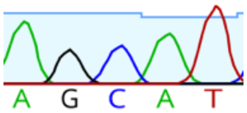	*2/*2	PM
24	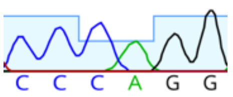	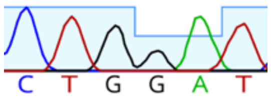	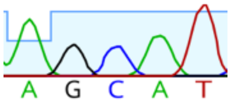	*2/*2	PM
25	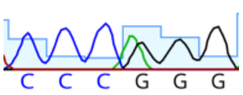	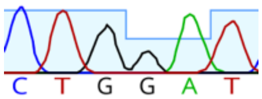	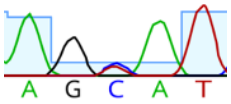	*2/*17	IM
26	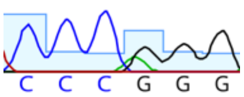	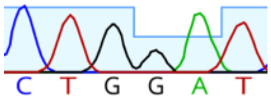	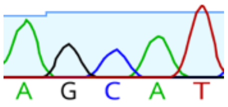	*2/*1	IM
27	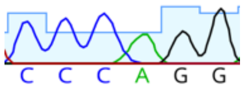	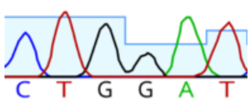	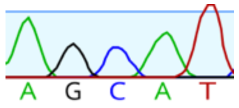	*2/*2	PM
28	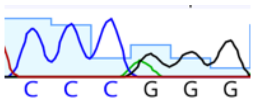	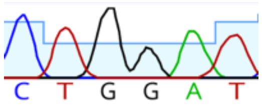	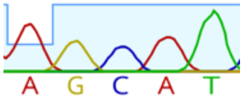	*2/*1	IM
29	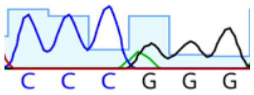	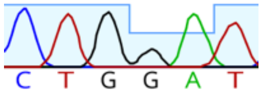	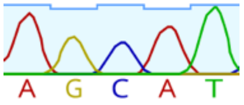	*2/*1	IM
30	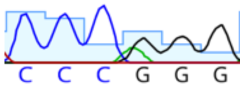	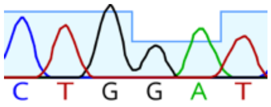	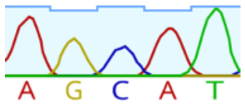	*2/*1	IM

Table S6. ‘Genetic code’ written by a battery of *DPYD* alleles for interpretation of DPYD drug responses

Drug responses	Genotype	*DPYD* Effect Allele#														Weighted-Sum
		rs56038477		rs112766203		rs3918290		rs55674432		rs67376798		rs1801266		rs72549304		
		c.1236G	c.1236A	c.2279C	c.2279T	c.1905+1G	c.1905+1A	c.2639G	c.2639T	c.2846A	c.2846T	c.703C	c.703T	c.1475C	c.1475T	
Poor	*8/*8	1	0	1	0	1	0	1	0	1	0	0	1	1	0	-4
	*8/HapB3	1	1	1	0	1	0	1	0	1	0	1	1	1	0	-6
	*8/c.2279C>T	1	0	1	1	1	0	1	0	1	0	1	1	1	0	-6
	*8/*2A	1	0	1	0	1	1	1	0	1	0	1	1	1	0	-12
	*8/c.2639G>T	1	0	1	0	1	0	1	1	1	0	1	1	1	0	-13
	*8/c.2846A>T	1	0	1	0	1	0	1	0	1	1	1	1	1	0	-6
	*8/c.1475C>T	1	0	1	0	1	0	1	0	1	0	1	1	1	1	-13
	HapB3/c.1475C>T	1	1	1	0	1	0	1	0	1	0	1	0	1	1	-6
	c.1475C>T/c.2279C>T	1	0	1	1	1	0	1	0	1	0	1	0	1	1	-6
	c.1475C>T/*2A	1	0	1	0	1	1	1	0	1	0	1	0	1	1	-12
	c.1475C>T/c.2639G>T	1	0	1	0	1	0	1	1	1	0	1	0	1	1	-13
	c.1475C>T/c.2846A>T	1	0	1	0	1	0	1	0	1	1	1	0	1	1	-6
	c.1475C>T/c.1475C>T	1	0	1	0	1	0	1	0	1	0	1	0	0	1	-4
	*2A/c.2279C>T	1	0	1	1	1	1	1	0	1	0	1	0	1	0	-5
	c.2279C>T/c.2639G>T	1	0	1	1	1	0	1	1	1	0	1	0	1	0	-6
	HapB3/*2A	1	1	1	0	1	1	1	0	1	0	1	0	1	0	-5
	HapB3/c.2639G>T	1	1	1	0	1	0	1	1	1	0	1	0	1	0	-6
	*2A/*2A	1	0	1	0	0	1	1	0	1	0	1	0	1	0	-3
	*2A/c.2639G>T	1	0	1	0	1	1	1	1	1	0	1	0	1	0	-12
	*2A/c.2846A>T	1	0	1	0	1	1	1	0	1	1	1	0	1	0	-5
	c.2639G>T/c.2639G>T	1	0	1	0	1	0	0	1	1	0	1	0	1	0	-4

	c.2639G>T/c.2846A>T	1	0	1	0	1	0	1	1	1	1	1	0	1	0	-6
Intermediate	HapB3/c.2279C>T	1	1	1	1	1	0	1	0	1	0	1	0	1	0	1
	c.2279C>T/c.2846A>T	1	0	1	1	1	0	1	0	1	1	1	0	1	0	1
	c.2279C>T/c.2279C>T	1	0	0	1	1	0	1	0	1	0	1	0	1	0	3
	HapB3/HapB3	0	1	1	0	1	0	1	0	1	0	1	0	1	0	3
	HapB3/c.2846A>T	1	1	1	0	1	0	1	0	1	1	1	0	1	0	1
	c.2846A>T/c.2846A>T	1	0	1	0	1	0	1	0	0	1	1	0	1	0	3
Normal	Two Alleles of normal function	1	0	1	0	1	0	1	0	1	0	1	0	1	0	7

Weights assigned to each allele: "1" for c.1236G, "-3" for c.1236A, "1" for c.2279C, "-3" for c.2279T, "1" for c.1905+1G, "-9" for c.1905+1A, "1" for c.2639G, "-10" for c.2639T, "1" for c.2846A, "-3" for c.2846T, "1" for c.703C, "-10" for c.703T, "1" for c.1475C, "-10" for c.1475T.

Table S7. 'Genetic codes' written by a battery of *NUDT15* and *TPMT* alleles

Drug response	Diplotype		*TPMT* Allele#								*NUDT15* Allele#			
			rs1800462		rs1142345		rs1800460		rs72552738		rs746071566		rs116855232	
	TPMT	*NUDT15*	238G	238C	719A	719C	460G	460A	395G	395A	GAGTCG (3)	GAGTCG (2)	7973C	7973T
Normal	*1/*1	*1/*1	1	0	1	0	1	0	1	0	1	0	1	0
TPMT intermediate, NUDT15 normal	*1/*2	*1/*1	1	1	1	0	1	0	1	0	1	0	1	0
	*1/*3B	*1/*1	1	0	1	0	1	1	1	0	1	0	1	0
	*1/*3C	*1/*1	1	0	1	1	1	0	1	0	1	0	1	0
	*1/*11	*1/*1	1	0	1	0	1	0	1	1	1	0	1	0
TPMT poor, NUDT15 normal	*2/*2	*1/*1	0	1	1	0	1	0	1	0	1	0	1	0
	*2/*3A	*1/*1	1	1	1	1	1	1	1	0	1	0	1	0
	*2/*3B	*1/*1	1	1	1	0	1	1	1	0	1	0	1	0
	*2/*3C	*1/*1	1	1	1	1	1	0	1	0	1	0	1	0
	*2/*11	*1/*1	1	1	1	0	1	0	1	1	1	0	1	0
	*3A/*3A	*1/*1	1	0	0	1	0	1	1	0	1	0	1	0
	*3A/*3B	*1/*1	1	0	1	1	0	1	1	0	1	0	1	0
	*3A/*3C	*1/*1	1	0	0	1	1	1	1	0	1	0	1	0
	*3A/*11	*1/*1	1	0	0	1	0	1	1	0	1	0	1	0
	*3B/*3B	*1/*1	1	0	1	0	0	1	1	0	1	0	1	0
	*3B/*3C	*1/*1	1	0	1	1	1	1	1	0	1	0	1	0
	*3B/*11	*1/*1	1	0	1	0	1	1	1	1	1	0	1	0
	*3C/*3C	*1/*1	1	0	0	1	1	0	1	0	1	0	1	0
	*3C/*11	*1/*1	1	0	1	1	1	0	1	1	1	0	1	0
	*11/*11	*1/*1	1	0	1	0	1	0	0	1	1	0	1	0
NUDT15 intermediate, TPMT normal	*1/*1	*1/*2	1	0	1	0	1	0	1	0	1	0	1	1
	*1/*1	*1/*3	1	0	1	0	1	0	1	0	1	0	1	1
	*1/*1	*1/*9	1	0	1	0	1	0	1	0	1	1	1	0
NUDT15 poor, TPMT normal	*1/*1	*2/*2	1	0	1	0	1	0	1	0	0	0	0	1
	*1/*1	*2/*3	1	0	1	0	1	0	1	0	1	0	0	1
	*1/*1	*2/*9	1	0	1	0	1	0	1	0	0	1	1	1
	*1/*1	*3/*3	1	0	1	0	1	0	1	0	1	0	0	1
	*1/*1	*3/*9	1	0	1	0	1	0	1	0	1	1	1	1
	*1/*1	*9/*9	1	0	1	0	1	0	1	0	0	1	1	0

weights assigned to each allele: "1.5" for c.238G, "-6" for c.238C, "1.5" for c.719A, "-6" for c.719C, "1.5" for c.460G, "-6" for c. 460A, "1" for c.395G, "-6" for c. 395A, "2" for c.50GAGTCG(3), "-9" for c.50 GAGTCG(2), "5" for c.7973C, "-8" for c.7973T.

Table S8. Directing the starting dose of thiopurines on the basis of weighted-sum concentrations in the parallel decoding circuit of *NUDT15* and *TPMT* ‘genetic code’

Recommended Dosing of Thiopurines[#]	Drug response	Diplotype		*TPMT* weighted-sum	*NUDT15* weighted-sum	X1+Y1 OR X2+Y2 <0?	X1+Y1< -1? OR X2+Y2 <-3?	>R3 (13)?
		TPMT	*NUDT15*	X1+Y1	X2+Y2			
Start with normal starting dose	TPMT and NUDT15 normal metabolizer	*1/*1	*1/*1	5.5	7	No	No	**Yes**
Start with reduced starting doses (30%-80% of normal dose)	TPMT intermediate, NUDT15 normal	*1/*2	*1/*1	-0.5	7	**Yes**	No	No
		*1/*3B	*1/*1	-0.5	7	**Yes**	No	No
		*1/*3C	*1/*1	-0.5	7	**Yes**	No	No
		*1/*11	*1/*1	-0.5	7	**Yes**	No	No
	NUDT15 intermediate metabolizer, TPMT normal metabolizer	*1/*1	*1/*2	5.5	-1	**Yes**	No	No
		*1/*1	*1/*3	5.5	-1	**Yes**	No	No
		*1/*1	*1/*9	5.5	-2	**Yes**	No	No
For malignancy, start with drastically reduced doses; For non-malignant conditions, consider alternative non-thiopurine immunosuppressive therapy	TPMT poor metabolizer, NUDT15 normal metabolizer	*2/*2	*1/*1	-2	7	**Yes**	**Yes**	No
		*2/*3A	*1/*1	-12.5	7	**Yes**	**Yes**	No
		*2/*3B	*1/*1	-6.5	7	**Yes**	**Yes**	No
		*2/*3C	*1/*1	-6.5	7	**Yes**	**Yes**	No
		*2/*11	*1/*1	-6.5	7	**Yes**	**Yes**	No
		*3A/*3A	*1/*1	-9.5	7	**Yes**	**Yes**	No
		*3A/*3B	*1/*1	-8	7	**Yes**	**Yes**	No
		*3A/*3C	*1/*1	-8	7	**Yes**	**Yes**	No
		*3A/*11	*1/*1	-9.5	7	**Yes**	**Yes**	No
		*3B/*3B	*1/*1	-2	7	**Yes**	**Yes**	No
		*3B/*3C	*1/*1	-6.5	7	**Yes**	**Yes**	No
		*3B/*11	*1/*1	-6.5	7	**Yes**	**Yes**	No
		*3C/*3C	*1/*1	-2	7	**Yes**	**Yes**	No
		*3C/*11	*1/*1	-6.5	7	**Yes**	**Yes**	No
		*11/*11	*1/*1	-1.5	7	**Yes**	**Yes**	No
	NUDT15 poor metabolizer, TPMT normal metabolizer	*1/*1	*2/*2	5.5	-8	**Yes**	**Yes**	No
		*1/*1	*2/*3	5.5	-6	**Yes**	**Yes**	No
		*1/*1	*2/*9	5.5	-12	**Yes**	**Yes**	No
		*1/*1	*3/*3	5.5	-6	**Yes**	**Yes**	No
		*1/*1	*3/*9	5.5	-10	**Yes**	**Yes**	No
		*1/*1	*9/*9	5.5	-4	**Yes**	**Yes**	No

The recommendations for adjusting starting doses of thiopurines based on *TPMT* and *NUDT15* genotypes were provided by Clinical Pharmacogenetics Implementation Consortium (CPIC) guideline[2].

Table S9. Genotypic data of four individuals from the 1000 Genomes Project phase 3

	HG00580 (M)	HG01256 (M)	HG00266 (F)	HG00138 (M)
rs13303010	G|A	A|G	G|A	A|A
rs2816938	T|T	T|T	T|T	T|T
rs3790844	G|A	A|A	A|G	A|A
rs1486134	T|G	T|G	G|T	G|T
rs9854771	G|G	A|G	A|G	A|A
rs2736098	C|C	T|C	C|T	C|C
rs35226131	C|C	C|C	C|C	C|C
rs401681	T|T	C|T	T|T	T|C
rs17688601	C|C	C|C	C|C	C|C
rs73328514	A|A	A|A	A|T	A|A
rs6971499	T|T	T|T	T|T	T|T
rs2941471	A|A	G|G	A|A	A|A
rs10094872	A|A	T|A	T|T	A|A
rs10991043	T|T	T|C	T|T	T|C
rs505922	C/T	C/C	C/C	C/T
rs9581943	A|G	A|G	G|A	G|G
rs9543325	C|T	T|C	T|T	T|C
rs7190458	A|G	G|G	G|G	G|G
rs4795218	G|G	G|G	G|G	A|G
rs7214041	C|T	C|T	C|C	C|C
rs1517037	C|T	T|C	C|T	C|C
rs16986825	C|T	C|C	C|T	T|C
Actual PRS	5.147	4.746	4.432	4.251
Circuit output[#]	5.113x	4.874x	4.511x	4.285x

Data shown were concentrations of PRS species outputted by the circuit at 4h. 1x=50nM.

Table S10. Comparison between our DNA-computing-based platform and Sanger sequencing for genetic testing and interpretation

	Sanger sequencing	Our platform
Multiplexing level	Low to medium	Low to high
Automation level	Medium	Highly automated with a robotic liquid handling system[3]
Turn-around time	14 to 18 hours for a single sample[4]	3.5 to 4 hours from raw samples to final results
Accuracy	High	High
Cost	≈$5 per kilobase (Increase with No. of loci)	≈$1 per sample (Changeless in multi-plex settings)
Target expandability	Low (DNA only)	High (compatible with non-DNA targets via bio- sensors)
Instrumentation requirements	Moderate (PCR and Sequencer)	Low (qPCR only)
Result analysis requirements	Need specialized software and experienced technicians	Little or no requirement for computer and laboratory specialists

Table S11. DNA sequences of gate strands

Probe name	Sequence (5' to 3')
CYP2C19-M_1 probe - top	ATGGGTGATTTTAAGTAATTTGTTATGTGTTCCTGGGAAATAATCAATGGGTATGG T
CYP2C19-M_1 probe - bottom	CATTGATTATTTCCCAGGAACACATAACAAATTACTTAAAATCACCCATCAACAACC ACATCTCACAC
CYP2C19-M_2 probe - top	ATGGGTGAAAAAAACTTGGCTTTACATGGATTCAGGGGAGTGCTTACAATCGTGA TAGAGGGTA
CYP2C19-M_2 probe - bottom	ATCACGATTGTAAGCACTCCCCTGAATCCATGTAAAGCCAAGTTTTTTTCACCCAT CAACAACCACATCTCACAC
CYP2C19-M_3 probe - top	TAGGTGGATTTTAAGTAATTTGTTATGTGTTCCCGGGAAATAATCAATAGTGGTG
CYP2C19-M_3 probe - bottom	TTGATTATTTCCCGGGAACACATAACAAATTACTTAAAATCCACCTACTATAATTCC ATCTCACAC
CYP2C19-M_4 probe - top	TAGGTGGAAAAAAACTTGGCTTTACATGGATCCAGGGGAGTGCTTACAATCGTGA TAAGGAGTG
CYP2C19-M_4 probe - bottom	ATCACGATTGTAAGCACTCCCCTGGATCCATGTAAAGCCAAGTTTTTTTCCACCTA CTATAATTCCATCTCACAC
CYP2C19-M_5 probe - top	TAGGTGGAAAATTTGTGTGTTCTGTTCTGAAAGTATCTCTGATGTAAGAAATAATG CGATGAGGT
CYP2C19-M_5 probe - bottom	GCATTATTTCTTACATCAGAGATACTTTCAGAACAGAACACACAAATTTTCCACCTA CTATAATTCCATCTCACAC
CYP2C19-F_1 probe	CATTGATTATTTCCCAGGAACACATAACAAATTACTTAAAATCACCCAT
CYP2C19-F_2 probe	ATCACGATTGTAAGCACTCCCCTGAATCCATGTAAAGCCAAGTTTTTTTCACCCAT
CYP2C19-F_3 probe	TTGATTATTTCCCGGGAACACATAACAAATTACTTAAAATCCACCTA
CYP2C19-F_4 probe	ATCACGATTGTAAGCACTCCCCTGGATCCATGTAAAGCCAAGTTTTTTTCCACCTA
CYP2C19-F_5 probe	GCATTATTTCTTACATCAGAGATACTTTCAGAACAGAACACACAAATTTTCCACCTA
CYP2C19-S_1 probe - top	ATCAACAACCACATCTCACACCTTCTTCATTCACCAACAT
CYP2C19-S_1 probe - bottom	AGGTGTGAGATGTGGTTGTTGATGGGTGA
CYP2C19-S_2 probe - top	TACTATAATTCCATCTCACACTATCCACTACACTCCTATC
CYP2C19-S_2 probe - bottom	TAGTGTGAGATGGAATTATAGTAGGTGGA
CYP2C19-Subtractgate-top	TATCCACTACACTCCTATCATGTTGGTGAATGAAGAAGGTGTGA
CYP2C19-Subtractgate-btm	CTTCTTCATTCACCAACATGATAGGAGTGTAGTGGATAGTGTGA
CYP2C19-T_1 probe - top	AGTATGTTGGTGAATGAAGAAGGTGTGAGA
CYP2C19-T_1 probe - bottom	ACACCTTCTTCATTCACCAACATACTCCTACCTACTCTCTCAACTCTA
CYP2C19-T_2 probe - top	GTAGATAGGAGTGTAGTGGATAGTGTGAGA
CYP2C19-T_2 probe - bottom	ACACTATCCACTACACTCCTATCTACCTAATCTCACCATATCTACTAA
Th_1 probe - top	TACTCCTACCTACTCTCTCAACTCTAACCCTCATTCAACTTCCAATTCA
CYP2C19-Th_1 probe - bottom	TAGAGTTGAGAGAGTAGGTAGGAGTATGTTGGTG
CYP2C19-Th_2 probe - top	ATCTACCTAATCTCACCATATCTACTAAACCACTCACTCATCACTT
CYP2C19-Th_2 probe - bottom	TTAGTAGATATGGTGAGATTAGGTAGATAGGAGTGTA
CYP2C19-Res_1 probe - top	CTCCTACCTACTCTCTCATAATACCATACACTCTACTAATCTCCA

CYP2C19-Res$_1$ probe - bottom	GTATTATGAGAGAGTAGGTAGGAGTATGTT
CYP2C19-F$_6$ probe	CTCCTACCTACTCTCTCATAATAC
CYP2C19-FAM Reporter-top	ACCATACACTCTACTAATCTCCA (3'-BHQ1)
CYP2C19-FAM Reporter - bottom	(5'-FAM) TGGAGATTAGTAGAGTGTATGGTATTATGAG
CYP2C19-Res$_2$ probe - top	ATCTACCTAATCTCACCATATCTACTAACTACCCTCATTACATACATTCCACA
CYP2C19-Res$_2$ probe - bottom	GGTAGTTAGTAGATATGGTGAGATTAGGTAGATAGGAG
CYP2C19-F$_7$ probe	ATCTACCTAATCTCACCATATCTACTAACTACC
CYP2C19-ROX Reporter - top	CCCTCATTACATACATTCCACA (3'-BHQ2)
CYP2C19-ROX Reporter - bottom	(5'-ROX) TGTGGAATGTATGTAATGAGGGTAGTTAGTA
CYP2C19-Res$_3$ probe - top	CTAACCCTCATTCAACTTCCAATTCATCCAACTATACCATCATCCACATCCAA
CYP2C19-Res$_3$ probe - bottom	TTGGATGAATTGGAAGTTGAATGAGGGTTAGAGTTG
CYP2C19-F$_8$ probe	CTAACCCTCATTCAACTTCCAATTCATCCAA
CYP2C19-VIC Reporter - top	CAACTATACCATCATCCACATCCAA (3'-BHQ1)
CYP2C19-VIC Reporter - bottom	(5'-VIC) TTGGATGTGGATGATGGTATAGTTGGATGAATTG
CYP2C19-Res$_4$ probe - top	TAAACCACTCACTCATCACTTACTTCCTCATCCACTACTACTCCAC
CYP2C19-Res$_4$ probe - bottom	GAAGTAAGTGATGAGTGAGTGGTTTAGTAGA
CYP2C19-F$_9$ probe	TAAACCACTCACTCATCACTTACTTC
CYP2C19-Cy5 Reporter - top	TTCCTCATCCACTACTACTCCAC (3'-BHQ3)
CYP2C19-Cy5 Reporter - bottom	(5'-Cy5) GTGGAGTAGTAGTGGATGAGGAAGTAAGTGA
CYP2C19-Filtergate1– top	TAACCCTCATTCAACTTCCAATTCA
CYP2C19-Filtergate1– btm	TGAATTGGAAGTTGAATGAGGGTTAGAGTTGAG
CYP2C19-Filtergate2– top	TAAACCACTCACTCATCACTT
CYP2C19-Filtergate2– btm	AAGTGATGAGTGAGTGGTTTAGTAGATATG
Mgate-DPYD-c.1236G-top	GGTAGTAGGCAGTTTGATTCGGACAGAGCAAGATGAAACAGGAAAAGAGAGAAG
Mgate-DPYD-c.1236G-bottom	TTTTCCTGTTTCATCTTGCTCTGTCCGAATCAAACTGCCTACTACCTAACCTATCTA CTACTTACAACACC-
Mgate-DPYD-c.1236A-top	GAGTAGAGGCAGTTTGATTCGGACAGAACAAGATGAAACAGGAAAAGAGTGTG
Mgate-DPYD-c.1236A-bottom	TTTTCCTGTTTCATCTTGTTCTGTCCGAATCAAACTGCCTCTACTCCTAATCCTAAC TCTCTCTACTCTCCACA
Mgate-DPYD-c.2279C-top	GTGAGAGATACCAGACAATCCTTCATATGTAGTTCGCTTTGCAATCAGTGTGTG
Mgate-DPYD-c.2279C-bottom	GATTGCAAAGCGAACTACATATGAAGGATTGTCTGGTATCTCTCACACATACTACT CTCACTACAACACC-
Mgate-DPYD-c.2279T-top	GAGTGAGATACCAGACAATCCTTCATATGTAATTCGCTTTGCAATCAGAGTGTG
Mgate-DPYD-c.2279T-bottom	GATTGCAAAGCGAATTACATATGAAGGATTGTCTGGTATCTCACTCACTAACTCAA CTCTCTCTACTCTCCACA-
Mgate-DPYD-c.1905+1G-top	GGTAGTAGTTATAGTTGTTAAATCACACTTACGTTGTCTGGAAAGTAGAGAGTG

Mgate-DPYD-c.1905+1G-bottom	ACTTTCCAGACAACGTAAGTGTGATTTAACAACTATAACTACTACCTAACCTATCTACTACTTACAACACC-
Mgate-DPYD-c.1905+1A-top	GAGTAGAGTTATAGTTGTTAAATCACACTTATGTTGTCTGGAAAGTCGAAGAGGT
Mgate-DPYD-c.1905+1A-bottom	GACTTTCCAGACAACATAAGTGTGATTTAACAACTATAACTCTACTCCTAATCCTAACTCTCTCTACTCTCCACA
Mgate-DPYD-c.2639G-top	GTGAGAGATTGAGCTGTTCGAGATAAGGTCCAAAACTAGTCAGATTCTAAAAGGTGGAGAGA
Mgate-DPYD-c.2639G-bottom	CCTTTTAGAATCTGACTAGTTTTGGACCTTATCTCGAACAGCTCAATCTCTCACACATACTACTCTCACTACAACACC
Mgate-DPYD-c.2639T-top	GAGTGAGACTTGAGCTGTTCGAGATAAGGTACAAAACTAGGCAGATTCTAAAATGAGAGTG
Mgate-DPYD-c.2639T-bottom	TTTTAGAATCTGCCTAGTTTTGTACCTTATCTCGAACAGCTCAAGTCTCACTCACTAACTCAACTCTCTCTACTCTCCACA-
Mgate-DPYD-c.2846A-top	GGTAGTAGACAGTTCATACACATTTCTTCATCAATCATAGCCACATAGAGAGG
Mgate-DPYD-c.2846A-bottom	TGTGGCTATGATTGATGAAGAAATGTGTATGAACTGTCTACTACCTAACCTATCTACTACTTACAACACC
Mgate-DPYD-c.2846T-top	GAGTAGAGACAGTTCATACACATTTCTTCAACAATCATAGCCACAAGAGAGAG
Mgate-DPYD-c.2846T-bottom	TGTGGCTATGATTGTTGAAGAAATGTGTATGAACTGTCTCTACTCCTAATCCTAACTCTCTCTACTCTCCACA
Mgate-DPYD-c.703C-top	GGTTGAGATTCACTACATCATACGAAGGAGCCGGAACTGAGGAATTTGAGAGATAGGAG
Mgate-DPYD-c.703C-bottom	TCTCAAATTCCTCAGTTCCGGCTCCTTCGTATGATGTAGTGAATCTCAACCAATTACAATCTTCAACAACACC
Mgate-DPYD-c.703T-top	GGTTGAGATTCACTACATCATACGAAGGAGCCAGAACTGAGGAATTTGAGAGAGTGAGT
Mgate-DPYD-c.703T-bottom	TCTCAAATTCCTCAGTTCTGGCTCCTTCGTATGATGTAGTGAATCTCAACCCATATCTCAACTCTCTCTTTCTCCACA-
Mgate-DPYD-c.1475C-top	GGTTGAGACTAACGCTACAGTGGAATCGGAGAATGATGGAAAGCTAGAGGTG
Mgate-DPYD-c.1475C-bottom	GCTTTCCATCATTCTCCGATTCCACTGTAGCGTTAGTCTCAACCAATTACAATCTTCAACAACACC-
Mgate-DPYD-c.1475T-top	GGTTGAGACTAACGCTACAGTGGAATTGGAGAATGATGGAAAGCTAGAGAGG
Mgate-DPYD-c.1475T-bottom	GCTTTCCATCATTCTCCAATTCCACTGTAGCGTTAGTCTCAACCCATATCTCAACTCTCTCTTTCTCCACA
Mgate-TPMT-c.238G-top	GTGATGTGAACTAAACTGTGTACCCGCTCTGCAAACCTTCATAAAATCATACAATTGGAGG
Mgate-TPMT-c.238G-bottom	TGTATGATTTTATGAAGGTTTGCAGAGCGGGTACACAGTTTAGTTCACATCACTCACTACATATTCACAACAACACC
Mgate-TPMT-c.238C-top	AGTGATGGAACTAAACTGTGTACCCGCTCTGGAAACCTTCATAAAATCATACAAGAGAGAG
Mgate-TPMT-c.238C-bottom	TGTATGATTTTATGAAGGTTTCCAGAGCGGGTACACAGTTTAGTTCCATCACTTATACATAATCTACACACATCTCC
Mgate-TPMT-c.719A-top	GTGATGTGTTGACTATCTTTTTGAAATGTTATATCTACTTACAGAAAATTAAATGTGGTGTGT
Mgate-TPMT-c.719A-bottom	CATTTAATTTTC TGTAAGTAGATATAACATTTCAAAAAGATAGTCAACACATCACTCACTACATATTCACAACAACACC
Mgate-TPMT-c.719G-top	AGTGATGGTTGACTATCTTTTTGAAATGTTATGTCTACTTACAGAAAATTAAATGTGGTGTGA
Mgate-TPMT-c.719G-bottom	CATTTAATTTTC TGTAAGTAGACATAACATTTCAAAAAGATAGTCAACCATCACTTATACATAATCTACACACATCTCC
Mgate-TPMT-c.460G-top	GTGATGTGTGCGATTACTTGTATTGATGGTAGACTAATGCTCCTCTATCCCAAATGTATGTGG
Mgate-TPMT-c.460G-bottom	ATTTGGGATAGAGGAGCATTAGTCTACCATCAATACAAGTAATCGCACACATCACTCACTACATATTCACAACAACACC
Mgate-TPMT-c.460A-top	GTTGTTGGTGCGATTACTTGTATTGATGGCAGACTAATGTTCCTCTATCCCAAATCAGTGTAGG

Mgate-TPMT-c.460A-bottom	GATTTGGGATAGAGGAACATTAGTCTGCCATCAATACAAGTAATCGCACCAACAA CTATACATAATCTACACACATCTCC
Mgate-TPMT-c.395G-top	GTGATGTGCCTGAGAAGATCAAAAATAATGCAACAGTACAATGAAATGGTCCCGG AAGTGATGG
Mgate-TPMT-c.395G-bottom	TCCGGGACCATTTCATTGTACTGTTGCATTATTTTTGATCTTCTCAGGCACATCACT CACTACATATTCACAACAACACC
Mgate-TPMT-c.395A-top	GTTGTTGGCCTGAGAAGATCAAAAATAATGCAATAGTACAATGAAATGGTCCCGG AGTGAGTGT
Mgate-TPMT-c.395A-bottom	TCCGGGACCATTTCATTGTACTATTGCATTATTTTTGATCTTCTCAGGCCAACAACT ATACATAATCTACACACATCTCC
Mgate-NUDT1c.GAGTCG(3)-top	AGGAGTGAGGGTGGCGGCCAGGAGTAGGAGTCGGAGTCAGGTGATG
Mgate-NUDT1c.GAGTCG(3)-bottom	GACTCCGACTCCTACTCCTGGCCGCCACCCTCACTCCTCAACACTCACATCTCAC AC
Mgate-NUDT1c.GAGTCG(2)-top	TGAGTGGAGGGTGGCGGCCAGGAGTAGGAGTCGTAGTGAGGTAGAGT
Mgate-NUDT1c.GAGTCG(2)-bottom	TCACTACGACTCCTACTCCTGGCCGCCACCCTCCACTCACTATAATTCCATCTCAC AC
Mgate-NUDT1c.7973C-top	AGGAGTGAACCGGCTTTTCTGGGGGCTGCGTTGTTTATAAGAATAAGGCTATGTG ATGTG
Mgate-NUDT1c.7973C-bottom	ATAGCCTTATTCTTATAAACAACGCAGCCCCCAGAAAAGCCGGTTCACTCCTCAAC ACTCACATCTCACAC
Mgate-NUDT1c.7973T-top	TGAGTGGAACCGGCTTTTCTGGGGGCTGTGTTGTTTATAAGAATAAGGCTATGTG GAGTGT
Mgate-NUDT1c.7973T-bottom	CATAGCCTTATTCTTATAAACAACACAGCCCCCAGAAAAGCCGGTTCCACTCACTA TAATTCCATCTCACAC
Sumgate-1-NUDT15-top	CTCAACACTCACATCTCACACCTTCTTCATTCACCAACAT
Sumgate-1-NUDT15-bottom	AGGTGTGAGATGTGAGTGTTGAGGAGTGA
Sumgate-2-NUDT15-top	CACTATAATTCCATCTCACACTATCCACTACACTCCTATC
Sumgate-2-NUDT15-bottom	TAGTGTGAGATGGAATTATAGTGAGTGGA
Sumgate-1-TPMT-top	ACTCACTACATATTCACAACAACACCTCCAACACCTCACCACCAACAT
Sumgate-1-TPMT-bottom	GAGGTGTTGTTGTGAATATGTAGTGAGTGATGTG
Sumgate-2-TPMT-top	ACTATACATAATCTACACACATCTCCTCCTTCACCTCACCACTCTCA
Sumgate-2-TPMT-bottom	GAGGAGATGTGTGTAGATTATGTATAGTTGTTGG
Sumgate-TPMT-top	CTTATACATAATCTACACACATCTCCTCCTTCACCTCACCACTCTCA
Sumgate-TPMT-bottom	GAGGAGATGTGTGTAGATTATGTATAAGTGATGG
Tgate-1-TPMT-top	AGTATGTTGGTGGTGAGGTGTTGGAGGT
Tgate-1-TPMT-bottom	CCAACACCTCACCACCAACATACTCCTACCTACTCTCTCAACTCTA
Tgate-2-TPMT-top	GTATGAGAGTGGTGAGGTGAAGGAGGAG
Tgate-2-TPMT-bottom	TCCTTCACCTCACCACTCTCATACACATCCTATCTTACCTCTCAC
Sub-TPMT-top	TCCTTCACCTCACCACTCTCAATGTTGGTGGTGAGGTGTTGGAGGTGTT
Sub-TPMT-bottom	TCCAACACCTCACCACCAACATTGAGAGTGGTGAGGTGAAGGAGGAGAT
R1-top	ATACACATCCTATCTTACCTCTCACTCATCTCACCACAACCTATCT
R1-bottom	GTGAGAGGTAAGATAGGATGTGTATGAGAGTGG
R2-top	TACTCCTACCTACTCTCTCAACTCTA

R2-bottom	TAGAGTTGAGAGAGTAGGTAGGAGTATGTTGGTG
R3-top	ATCTACCTAATCTCACCATATCTACTAAACCACTCACTCATCACTT
R3-bottom	TTAGTAGATATGGTGAGATTAGGTAGATAGGAGTGTA
Res1-TPMT-top	ATACACATCCTATCTTACCTCTCACCACACCCTTCATCCTCACAC
Res1-TPMT-bottom	GTGTGGTGAGAGGTAAGATAGGATGTGTATGAGAG
Res2-TPMT-top	CACTCATCTCACCACAACCTATCTTCCACCACCTCACACTCTCACTTCC
Res2-TPMT-bottom	GTGGAAGATAGGTTGTGGTGAGATGAGTGAGAGG
Res1-NUDT15-top	ATCTACCTAATCTCACCATATCTACTAACTCACCATTATCCATCTCACCTT
Res1-NUDT15-bottom	GTGAGTTAGTAGATATGGTGAGATTAGGTAGATAGGAG
Res2-NUDT15-top	TAAACCACTCACTCATCACTTCATACTCCACTATCTTACCTCAC
Res2-NUDT15-bottom	AGTATGAAGTGATGAGTGAGTGGTTTAGTAGAT
Res-NUDT15/TPMT-top	ATACTCCTACCTACTCTCTCAACTCTATCACCCACTTACTTCTCTCTACTTCT
Res-NUDT15/TPMT-bottom	GGTGATAGAGTTGAGAGAGTAGGTAGGAGTATGTTGG
Filter-R1-top	ACTCATCTCACCACAACCTATCT
Filter-R1-bottom	AGATAGGTTGTGGTGAGATGAGTGAGAGGTA
Filter-R2-top	TAAACCACTCACTCATCACTT
Filter-R2-bottom	AAGTGATGAGTGAGTGGTTTAGTAGATATG
Fuel-Res1-TPMT	ATACACATCCTATCTTACCTCTCACCACAC
Fuel-Res2-TPMT	CACTCATCTCACCACAACCTATCTTCCAC
Fuel-Res1-NUDT15	ATCTACCTAATCTCACCATATCTACTAACTCAC
Fuel-Res2-NUDT15	TAAACCACTCACTCATCACTTCATACT
Fuel-Res-NUDT15/TPMT	ATACTCCTACCTACTCTCTCAACTCTATCACC
Rep-FAM-TPMT-top	ACCCTTCATCCTCACAC (3’-BHQ1)
Rep-FAM-TPMT-bottom	(5’-FAM) GTGTGAGGATGAAGGGTGTGGTGAG
Rep-FAM-NUDT15-top	ACCATTATCCATCTCACCTT (3’-BHQ1)
Rep-FAM-NUDT15-bottom	(5’-FAM) AAGGTGAGATGGATAATGGTGAGTTAGT
Rep-VIC-TPMT-top	ACCACCTCACACTCTCACTTCC (3’-BHQ1)
Rep-VIC-TPMT-bottom	(5’-VIC) GGAAGTGAGAGTGTGAGGTGGTGGAAGATA
Rep-VIC-NUDT15-top	CTCCACTATCTTACCTCAC (3’-BHQ1)
Rep-VIC-NUDT15-bottom	(5’-VIC) GTGAGGTAAGATAGTGGAGTATGAAGTG
Rep-ROX-NUDT15/TPMT-top	CCCACTTACTTCTCTCTACTTCT (3’-BHQ2)
Rep-ROX-NUDT15/TPMT-bottom	(5’-ROX) AGAAGTAGAGAGAAGTAAGTGGGTGATAGAG
Sum1-DPYD-top	CTAACCTATCTACTACTTACAACACCTATCCACTACACTCCTATC
Sum1-DPYD-btm	TAGGTGTTGTAAGTAGTAGATAGGTTAGGTAGTAG

Sum2-DPYD-top	ACACATACTACTCTCACTACAACACCTATCCACTACACTCCTATC
Sum2-DPYD-btm	TAGGTGTTGTAGTGAGAGTAGTATGTGTGAGAGA
Sum3-DPYD-top	CAATTACAATCTTCAACAACACCTATCCACTACACTCCTATC
Sum3-DPYD-btm	TAGGTGTTGTTGAAGATTGTAATTGGTTGAGA
Sum4-DPYD-top	TCCTAATCCTAACTCTCTCTACTCTCCACACTTCTTCATTCACCAACAT
Sum4-DPYD-btm	AGTGTGGAGAGTAGAGAGAGTTAGGATTAGGAGTAGAG
Sum5-DPYD-top	TCACTAACTCAACTCTCTCTACTCTCCACACTTCTTCATTCACCAACAT
Sum5-DPYD-btm	AGTGTGGAGAGTAGAGAGAGTTGAGTTAGTGAGTGAGA
Sum6-DPYD-top	CCATATCTCAACTCTCTCTTTCTCCACACTTCTTCATTCACCAACAT
Sum6-DPYD-btm	AGTGTGGAGAAAGAGAGAGTTGAGATATGGGTTGAGA
Sub-DPYD-top	TATCCACTACACTCCTATCATGTTGGTGAATGAAGAAGTGTGGA
Sub-DPYD-btm	CTTCTTCATTCACCAACATGATAGGAGTGTAGTGGATAGGTGTT
T1-DPYD-top	AGTATGTTGGTGAATGAAGAAGTGTG
T1-DPYD-bottom	CTTCTTCATTCACCAACATACTCCTACCTACTCTCTCAACTCTA
T2-DPYD-top	GTAGATAGGAGTGTAGTGGATAGGTGT
T2-DPYD-bottom	CTATCCACTACACTCCTATCTACCTAATCTCACCATATCTACTAA
Filter-T1-top	CTCCTACCTACTCTCTCAACTCTA
Filter-T1-bottom	TAGAGTTGAGAGAGTAGGTAGGAGTATGTTGG
rs13303010-Ef-M-top	GAAGAGGACCACAGGGAGGCAAAATCGGGAGCAAGAAAAGACCAGAGAGTG
rs13303010-Ef-M-btm	GGTCTTTTCTTGCTCCCGATTTTGCCTCCCTGTGGTCCTCTTCCTTCTCTCTATCC CTCAC
rs13303010-Ot-M-top	GATGGAGACCACAGGGAGGCAAAATCAGGAGCAAGAAAAGACCAGAGGAGT
rs13303010-Ot-M-btm	GGTCTTTTCTTGCTCCTGATTTTGCCTCCCTGTGGTCTCCATCCTCTCCATCCTCT TCCTA
rs13303010-Ann-top	TCCTTCTCTCTATCCCTCACTAGGAAGAGGATGGAGAGGATGGAGA
rs13303010-Ann-btm	TCCTCTCCATCCTCTTCCTAGTGAGGGATAGAGAGAAGGAAGAGGA
rs13303010-Trans-top	GTGTGTGAGGGATAGAGAGAAGGAAGAGGA
rs13303010-Trans-btm	CTTCCTTCTCTCTATCCCTCACACACTATCCTAACTCACCACAC
in-rs13303010-Ef	CACTCTCTGGTCTTTTCTTGCTCCCGATTTTGCCTCCCTGTGG
in-rs13303010-Ot	ACTCCTCTGGTCTTTTCTTGCTCCTGATTTTGCCTCCCTGTGG
fuel-rs13303010-Ef	GGTCTTTTCTTGCTCCCGATTTTGCCTCCCTGTGGTCCTCTTC
fuel-rs13303010-Ot	GGTCTTTTCTTGCTCCTGATTTTGCCTCCCTGTGGTCTCCATC
rs2816938-Ef-M-top	GAGTGTGTGGCCTCGCAAATTGATGGGATTACAGGTGATAGCTACTGGAGTGAGT
rs2816938-Ef-M-btm	CAGTAGCTATCACCTGTAATCCCATCAATTTGCGAGGCCACACACTCCACACACA CACTACTCAC
rs2816938-Ot-M-top	TGTGTGTGGGCCTCGCAAATTGATGGGAATACAGGTGATAGCTACTGGTGATGAG
rs2816938-Ot-M-btm	CAGTAGCTATCACCTGTATTCCCATCAATTTGCGAGGCCCACACACACACTCCAC ATACACCACA

rs2816938-Ann-top	TCCACACACACACTACTCACTGTGGTGTATGTGGAGTGTGTGTGTG
rs2816938-Ann-btm	CACACTCCACATACACCACAGTGAGTAGTGTGTGTGTGGAGTGTGT
rs2816938-Trans-top	TGTGTGAGTAGTGTGTGTGTGGAGTGTGT
rs2816938-Trans-btm	ACTCCACACACACACTACTCACACACTATCCTAACTCACCACAC
in-rs2816938-Ef	ACTCACTCCAGTAGCTATCACCTGTAATCCCATCAATTTGCGAGGCC
in-rs2816938-Ot	CTCATCACCAGTAGCTATCACCTGTATTCCCATCAATTTGCGAGGCC
fuel-rs2816938-Ef	CAGTAGCTATCACCTGTAATCCCATCAATTTGCGAGGCCACACACTC
fuel-rs2816938-Ot	CAGTAGCTATCACCTGTATTCCCATCAATTTGCGAGGCCCACACACA
rs3790844-Ef-M-top	GATTGGAGAGGTCACTAAAATTGGAAAGTCTGTCGCTGGATGATG
rs3790844-Ef-M-btm	AGCGACAGACTTTCCAATTTTAGTGACCTCTCCAATCCTCTACTCCATCTCTCAC
rs3790844-Ot-M-top	GATGAGAGAGGTCACTAAAATTGGGAAGTCTGTCGCTGAGAGTAG
rs3790844-Ot-M-btm	AGCGACAGACTTCCCAATTTTAGTGACCTCTCTCATCCATCAACTCCACACCACT
rs3790844-Ann-top	TCCTCTACTCCATCTCTCACAGTGGTGTGGAGTTGATGGATGAGAG
rs3790844-Ann-btm	TCCATCAACTCCACACCACTGTGAGAGATGGAGTAGAGGATTGGAG
rs3790844-Trans-top	GTGTGTGAGAGATGGAGTAGAGGATTGGA
rs3790844-Trans-btm	ATCCTCTACTCCATCTCTCACACACTATCCTAACTCACCACAC
in-rs3790844-Ef	CATCATCCAGCGACAGACTTTCCAATTTTAGTGACCT
in-rs3790844-Ot	CTACTCTCAGCGACAGACTTCCCAATTTTAGTGACCT
fuel-rs3790844-Ef	AGCGACAGACTTTCCAATTTTAGTGACCTCTCCAATC
fuel-rs3790844-Ot	AGCGACAGACTTCCCAATTTTAGTGACCTCTCTCATC
rs1486134-Ef-M-top	GATGTGTGACTCGGACTCTAGGAACATTGGGGTTGCTTACTGTGAGATG
rs1486134-Ef-M-btm	AGTAAGCAACCCCAATGTTCCTAGAGTCCGAGTCACACATCCACACACTTCTACCTCAC
rs1486134-Ot-M-top	GATGTGTGACTCGGACTCTAGGAACATTTGGGTTGCTTACTGGAGGATTG
rs1486134-Ot-M-btm	CAGTAAGCAACCCAAATGTTCCTAGAGTCCGAGTCACACATCCATCTTCTCACATCCACC
rs1486134-Ann-top	TCCACACACTTCTACCTCACGGTGGATGTGAGAAGATGGATGTGTG
rs1486134-Ann-btm	TCCATCTTCTCACATCCACCGTGAGGTAGAAGTGTGTGGATGTGTG
rs1486134-Trans-top	TGTGTGAGGTAGAAGTGTGTGGATGTGTG
rs1486134-Trans-btm	CATCCACACACTTCTACCTCACACACTATCCTAACTCACCACAC
in-rs1486134-Ef	CATCTCACAGTAAGCAACCCCAATGTTCCTAGAGTCCGAGT
in-rs1486134-Ot	CAATCCTCCAGTAAGCAACCCAAATGTTCCTAGAGTCCGAGT
fuel-rs1486134-Ef	AGTAAGCAACCCCAATGTTCCTAGAGTCCGAGTCACACATC
fuel-rs1486134-Ot	CAGTAAGCAACCCAAATGTTCCTAGAGTCCGAGTCACACATC
rs9854771-Ef-M-top	GGAGAGTTCAACACAGCTGCCCCATTCTAAGTAATATAAACTAATTGAGATGG
rs9854771-Ef-M-btm	ATTAGTTTATATTACTTAGAATGGGGCAGCTGTGTTGAACTCTCCTACTCTCTCACTCCTCAC

rs9854771-Ot-M-top	GGGTAGTTCAACACAGCTGCTCCATTCTAAGTAATATAAACTAATAGGGTAGAA
rs9854771-Ot-M-btm	TATTAGTTTATATTACTTAGAATGGAGCAGCTGTGTTGAACTACCCTCTTATATCTC TCCCTCC
rs9854771-Ann-top	CTACTCTCTCACTCCTCACGGAGGGAGAGATATAAGAGGGTAGT
rs9854771-Ann-btm	CTCTTATATCTCTCCCTCCGTGAGGAGTGAGAGAGTAGGAGAGT
rs9854771-Trans-top	TGTGTGAGGAGTGAGAGAGTAGGAGAGT
rs9854771-Trans-btm	TCCTACTCTCTCACTCCTCACACACTATCCTAACTCACCACAC
in- rs9854771-Ef	CCATCTCAATTAGTTTATATTACTTAGAATGGGGCAGCTGTGTTG
in- rs9854771-Ot	TTCTACCCTATTAGTTTATATTACTTAGAATGGAGCAGCTGTGTTG
fuel-rs9854771-Ef	ATTAGTTTATATTACTTAGAATGGGGCAGCTGTGTTGAACTCTCC
fuel-rs9854771-Ot	TATTAGTTTATATTACTTAGAATGGAGCAGCTGTGTTGAACTACCC
rs2736098-Ef-M-top	GAGAGTGTGATGGGGGGCTCGCGTGGTTCTGGTGTGATGG
rs2736098-Ef-M-btm	CCAGAACCACGCGAGCCCCCCATCACACTCTCCATTCCTACCATCCTCAC
rs2736098-Ot-M-top	GAGGATGTGATGGGGGGCTTGCTTGGTTCTGGCGTGAGTGAG
rs2736098-Ot-M-btm	CGCCAGAACCAAGCAAGCCCCCCATCACATCCTCCAACTCTTCATCCCACTC
rs2736098-Ann-top	TCCATTCCTACCATCCTCACGAGTGGGATGAAGAGTTGGAGGATGT
rs2736098-Ann-btm	TCCAACTCTTCATCCCACTCGTGAGGATGGTAGGAATGGAGAGTGT
rs2736098-Trans-top	TGTGTGAGGATGGTAGGAATGGAGAGTGT
rs2736098-Trans-btm	TCTCCATTCCTACCATCCTCACACACTATCCTAACTCACCACAC
in-rs2736098-Ef	CCATCACACCAGAACCACGCGAGCCCCCCATC
in-rs2736098-Ot	CTCACTCACGCCAGAACCAAGCAAGCCCCCCATC
fuel-rs2736098-Ef	CCAGAACCACGCGAGCCCCCCATCACACTCTC
fuel-rs2736098-Ot	CGCCAGAACCAAGCAAGCCCCCCATCACATCCTC
rs35226131-Ef-M-top	TGTGAGTGGTCCTGTGTGTCTGTGCCTGCGAATCCATTGGAGAGTGTG
rs35226131-Ef-M-btm	CCAATGGATTCGCAGGCACAGACACACAGGACCACTCACACACTCAACCATACCT CAC
rs35226131-Ot-M-top	GTGTGTGTGTCCTGTGTGTCTGTGCTTGCGAATCCATTGGAGAGAGTG
rs35226131-Ot-M-btm	CCAATGGATTCGCAAGCACAGACACACAGGACACACACACCACTTCACTCACTCA CCT
rs35226131-Ann-top	CACACTCAACCATACCTCACAGGTGAGTGAGTGAAGTGGTGTGTGT
rs35226131-Ann-btm	ACCACTTCACTCACTCACCTGTGAGGTATGGTTGAGTGTGTGAGTG
rs35226131-Trans-top	TGTGTGAGGTATGGTTGAGTGTGTGAGTG
rs35226131-Trans-btm	CACACACTCAACCATACCTCACACACTATCCTAACTCACCACAC
in-rs35226131-Ef	CACACTCTCCAATGGATTCGCAGGCACAGACACACAGGAC
in-rs35226131-Ot	CACTCTCTCCAATGGATTCGCAAGCACAGACACACAGGAC
fuel-rs35226131-Ef	CCAATGGATTCGCAGGCACAGACACACAGGACCACTCACA
fuel-rs35226131-Ot	CCAATGGATTCGCAAGCACAGACACACAGGACACACACAC

rs401681-Ef-M-top	TGGTGAGTGCTATCCAGATAAATTCAGAGTCATTCATAGTGTTAAGCAGCTTGGTG AGT
rs401681-Ef-M-btm	AGCTGCTTAACACTATGAATGACTCTGAATTTATCTGGATAGCACTCACCACTTAC ATACCTCCCTCAC
rs401681-Ot-M-top	TGAGGTGTGCTATCCAGATAAATTCAGAGTCCTTCATTGTGTTAAGCAGCTTTTGT GGTGT
rs401681-Ot-M-btm	AAAGCTGCTTAACACAATGAAGGACTCTGAATTTATCTGGATAGCACACCTCACTA CACTCTACTCACTCC
rs401681-Ann-top	CACTTACATACCTCCCTCACGGAGTGAGTAGAGTGTAGTGAGGTGT
rs401681-Ann-btm	CACTACACTCTACTCACTCCGTGAGGGAGGTATGTAAGTGGTGAGT
rs401681-Trans-top	TGTGTGAGGGAGGTATGTAAGTGGTGAGT
rs401681-Trans-btm	ACCACTTACATACCTCCCTCACACACTATCCTAACTCACCACAC
in-rs401681-Ef	ACTCACCAAGCTGCTTAACACTATGAATGACTCTGAATTTATCTGGATAGC
in-rs401681-Ot	ACACCACAAAAGCTGCTTAACACAATGAAGGACTCTGAATTTATCTGGATAGC
fuel-rs401681-Ef	AGCTGCTTAACACTATGAATGACTCTGAATTTATCTGGATAGCACTCACCA
fuel-rs401681-Ot	AAAGCTGCTTAACACAATGAAGGACTCTGAATTTATCTGGATAGCACACCTCA
rs17688601-Ef-M-top	AGGTGAGTTGTGGTAAGCATAGTTAACATCCTGACGAGTGAAAATGAATTGGTGG TA
rs17688601-Ef-M-btm	ATTCATTTTCACTCGTCAGGATGTTAACTATGCTTACCACAACTCACCTCATCTCCA CTTCACTCAC
rs17688601-Ot-M-top	ATGGTAGATGTGGTAAGCATAGTTAAAATCCTGACGAGTGAAAATGAATGTGGAG TA
rs17688601-Ot-M-btm	ATTCATTTTCACTCGTCAGGATTTTAACTATGCTTACCACATCTACCATCACCAATA CTCCTCATCC
rs17688601-Ann-top	CTCATCTCCACTTCACTCACGGATGAGGAGTATTGGTGATGGTAGA
rs17688601-Ann-btm	ATCACCAATACTCCTCATCCGTGAGTGAAGTGGAGATGAGGTGAGT
rs17688601-Trans-top	TGTGTGAGTGAAGTGGAGATGAGGTGAGT
rs17688601-Trans-btm	ACCTCATCTCCACTTCACTCACACACTATCCTAACTCACCACAC
in-rs17688601-Ef	TACCACCAATTCATTTTCACTCGTCAGGATGTTAACTATGCTTACCACA
in-rs17688601-Ot	TACTCCACATTCATTTTCACTCGTCAGGATTTTAACTATGCTTACCACA
fuel-rs17688601-Ef	ATTCATTTTCACTCGTCAGGATGTTAACTATGCTTACCACAACTCACCT
fuel-rs17688601-Ot	ATTCATTTTCACTCGTCAGGATTTTAACTATGCTTACCACATCTACCAT
rs73328514-Ef-M-top	AGGTGTGTATCTGTGGAGCGGGATAATGATGTCTCTCTTGTGATTGGTGGT
rs73328514-Ef-M-btm	TCACAAGAGAGACATCATTATCCCGCTCCACAGATACACACCTCACCATTACCATC CTCAC
rs73328514-Ot-M-top	TGTGGTGTATCTGTGGAGCGGGATTATGATGTCTCTCTTGTGATGTGGTGT
rs73328514-Ot-M-btm	TCACAAGAGAGACATCATAATCCCGCTCCACAGATACACCACACAATCCACACTT CCACCT
rs73328514-Ann-top	CTCACCATTACCATCCTCACAGGTGGAAGTGTGGATTGTGTGGTGT
rs73328514-Ann-btm	CACAATCCACACTTCCACCTGTGAGGATGGTAATGGTGAGGTGTGT
rs73328514-Trans-top	TGTGTGAGGATGGTAATGGTGAGGTGTGT
rs73328514-Trans-btm	ACCTCACCATTACCATCCTCACACACTATCCTAACTCACCACAC

in-rs73328514-Ef	ACCACCAATCACAAGAGAGACATCATTATCCCGCTCCACAGAT
in-rs73328514-Ot	ACACCACATCACAAGAGAGACATCATAATCCCGCTCCACAGAT
fuel-rs73328514-Ef	TCACAAGAGAGACATCATTATCCCGCTCCACAGATACACACCT
fuel-rs73328514-Ot	TCACAAGAGAGACATCATAATCCCGCTCCACAGATACACCACA
rs6971499-Ef-M-top	GAGTGAGTCAGTTCTAGGTAAATCTTTGATCATGTTAAAATATATAAGTTTCTGGG AGGGTGTGTGG
rs6971499-Ef-M-btm	CCTCCCAGAAACTTATATATTTTAACATGATCAAAGATTTACCTAGAACTGACTCAC TCCACACTTCACCATCTCAC
rs6971499-Ot-M-top	AGGTGTGTCAGTTCTAGGTAAATCTTTGATCATGCTAAAATATATAAGTTTCTGGG AGGAGGTGAGT
rs6971499-Ot-M-btm	CCTCCCAGAAACTTATATATTTTAGCATGATCAAAGATTTACCTAGAACTGACACA CCTCAACTCAACCATCCACCA
rs6971499-Ann-top	TCCACACTTCACCATCTCACTGGTGGATGGTTGAGTTGAGGTGTGT
rs6971499-Ann-btm	CTCAACTCAACCATCCACCAGTGAGATGGTGAAGTGTGGAGTGAGT
rs6971499-Trans-top	TGTGTGAGATGGTGAAGTGTGGAGTGAGT
rs6971499-Trans-btm	ACTCCACACTTCACCATCTCACACACTATCCTAACTCACCACAC
in-rs6971499-Ef	CCACACACCCTCCCAGAAACTTATATATTTTAACATGATCAAAGATTTACCTAGAAC TG
in-rs6971499-Ot	ACTCACCTCCTCCCAGAAACTTATATATTTTAGCATGATCAAAGATTTACCTAGAAC TG
fuel-rs6971499-Ef	CCTCCCAGAAACTTATATATTTTAACATGATCAAAGATTTACCTAGAACTGACTCAC TC
fuel-rs6971499-Ot	CCTCCCAGAAACTTATATATTTTAGCATGATCAAAGATTTACCTAGAACTGACACA CCT
rs2941471-Ef-M-top	AGGTGAGTCTTTAGGGTTTGTATTATAGTCTTTAGTGAATAAAATGCTAGTGGAGA
rs2941471-Ef-M-btm	AGCATTTTATTCACTAAAGACTATAATACAAACCCTAAAGACTCACCTCACATCACA CAACCTCAC
rs2941471-Ot-M-top	AGGTGAGTCTTTAGGGTTTGTATTGTAGTCTTTAGTGAATAAAATGCTGAGGTTGT
rs2941471-Ot-M-btm	AGCATTTTATTCACTAAAGACTACAATACAAACCCTAAAGACTCACCTCACTCTCC ATCACCACAA
rs2941471-Ann-top	CTCACATCACACAACCTCACTTGTGGTGATGGAGAGTGAGGTGAGT
rs2941471-Ann-btm	CTCACTCTCCATCACCACAAGTGAGGTTGTGTGATGTGAGGTGAGT
rs2941471-Trans-top	TGTGTGAGGTTGTGTGATGTGAGGTGAGT
rs2941471-Trans-btm	ACCTCACATCACACAACCTCACACACTATCCTAACTCACCACAC
in-rs2941471-Ef	TCTCCACTAGCATTTTATTCACTAAAGACTATAATACAAACCCTAAAG
in-rs2941471-Ot	ACAACCTCAGCATTTTATTCACTAAAGACTACAATACAAACCCTAAAG
fuel-rs2941471-Ef	AGCATTTTATTCACTAAAGACTATAATACAAACCCTAAAGACTCACCT
fuel-rs2941471-Ot	AGCATTTTATTCACTAAAGACTACAATACAAACCCTAAAGACTCACCT
rs10094872-Ef-M-top	AGGTGAGTATAAGTGGCTGTGATTTTATTTGTTCTCTATTGGTCTGTATGGTGGT
rs10094872-Ef-M-btm	ACAGACCAATAGAGAACAAATAAAATCACAGCCACTTATACTCACCTCACACTACA CAACCTCAC
rs10094872-Ot-M-top	TGGGTAGTATAAGTGGCTGTGATTTTAATTGTTCTCTATTGGTCTGTTGTGGTGT
rs10094872-Ot-M-btm	ACAGACCAATAGAGAACAATTAAAATCACAGCCACTTATACTACCCACACCTCTTA CCTTCACTC

rs10094872-Ann-top	CTCACACTACACAACCTCACGAGTGAAGGTAAGAGGTGTGGGTAGT
rs10094872-Ann-btm	CACACCTCTTACCTTCACTCGTGAGGTTGTGTAGTGTGAGGTGAGT
rs10094872-Trans-top	TGTGTGAGGTTGTGTAGTGTGAGGTGAGT
rs10094872-Trans-btm	ACCTCACACTACACAACCTCACACACTATCCTAACTCACCACAC
in-rs10094872-Ef	ACCACCATACAGACCAATAGAGAACAAATAAAATCACAGCCACTTAT
in-rs10094872-Ot	ACACCACAACAGACCAATAGAGAACAATTAAAATCACAGCCACTTAT
fuel-rs10094872-Ef	ACAGACCAATAGAGAACAAATAAAATCACAGCCACTTATACTCACCT
fuel-rs10094872-Ot	ACAGACCAATAGAGAACAATTAAAATCACAGCCACTTATACTACCCA
rs10991043-Ef-M-top	TTGTGTGATTTAACCTTTCTCCTACCTGCATACTCCTTTGTGTGA
rs10991043-Ef-M-btm	AGGAGTATGCAGGTAGGAGAAAGGTTAAATCACACAACACAACACACACACTCAC
rs10991043-Ot-M-top	TAGTGTGATTTAACCTTTCTCCTATCTGCATACTCCTTTGTTGTGA
rs10991043-Ot-M-btm	AAGGAGTATGCAGATAGGAGAAAGGTTAAATCACACTACACTCAACTACCACACCA
rs10991043-Ann-top	AACACAACACACACACTCACTGGTGTGGTAGTTGAGTGTAGTGTGA
rs10991043-Ann-btm	TACACTCAACTACCACACCAGTGAGTGTGTGTGTTGTGTTGTGTGA
rs10991043-Trans-top	TGTGTGAGTGTGTGTGTTGTGTTGTGTGA
rs10991043-Trans-btm	ACAACACAACACACACACTCACACACTATCCTAACTCACCACAC
in-rs10991043-Ef	TCACACAAAGGAGTATGCAGGTAGGAGAAAGGTTAAA
in-rs10991043-Ot	TCACAACAAAGGAGTATGCAGATAGGAGAAAGGTTAAA
fuel-rs10991043-Ef	AGGAGTATGCAGGTAGGAGAAAGGTTAAATCACACAA
fuel-rs10991043-Ot	AAGGAGTATGCAGATAGGAGAAAGGTTAAATCACACTA
rs505922-Ef-M-top	GTGTGTGTCAGCTGTACCTTTCATGTGCGATTTATTGTATAGGATGGAT
rs505922-Ef-M-btm	TATACAATAAATCGCACATGAAAGGTACAGCTGACACACACACACTTCCACATCCTCAC
rs505922-Ot-M-top	GAGAGTGTCAGCTGTACCTTTTATGTGCGATTTATTGTATAGTGAGTGT
rs505922-Ot-M-btm	TATACAATAAATCGCACATAAAAGGTACAGCTGACACTCTCCACACCCAACTCACATAC
rs505922-Ann-top	ACACACTTCCACATCCTCACGTATGTGAGTTGGGTGTGGAGAGTGT
rs505922-Ann-btm	TCCACACCCAACTCACATACGTGAGGATGTGGAAGTGTGTGTGTGT
rs505922-Trans-top	TGTGTGAGGATGTGGAAGTGTGTGTGTGT
rs505922-Trans-btm	ACACACACTTCCACATCCTCACACACTATCCTAACTCACCACAC
in-rs505922-Ef	ATCCATCCTATACAATAAATCGCACATGAAAGGTACAGCTG
in-rs505922-Ot	ACACTCACTATACAATAAATCGCACATAAAAGGTACAGCTG
fuel-rs505922-Ef	TATACAATAAATCGCACATGAAAGGTACAGCTGACACACAC
fuel-rs505922-Ot	TATACAATAAATCGCACATAAAAGGTACAGCTGACACTCTC
rs9581943-Ef-M-top	GAGTAGGTGGAACAAAAGCAGATGATCGCGGGTACTTGGGGGATGTGT
rs9581943-Ef-M-btm	CCCAAGTACCCGCGATCATCTGCTTTTGTTCCACCTACTCCATTCTCCTACCTCTCAC

rs9581943-Ot-M-top	GTGTGAGTGGAACAAAAGCAGGTGATCGCGGGTACTTGGGGTGTGAGT
rs9581943-Ot-M-btm	CCCAAGTACCCGCGATCACCTGCTTTTGTTCCACTCACACCATCACCACTACACACTC
rs9581943-Ann-top	TCCATTCTCCTACCTCTCACGAGTGTGTAGTGGTGATGGTGTGAGT
rs9581943-Ann-btm	ACCATCACCACTACACACTCGTGAGAGGTAGGAGAATGGAGTAGGT
rs9581943-Trans-top	TGTGTGAGAGGTAGGAGAATGGAGTAGGT
rs9581943-Trans-btm	ACTCCATTCTCCTACCTCTCACACACTATCCTAACTCACCACAC
in-rs9581943-Ef	ACACATCCCCCAAGTACCCGCGATCATCTGCTTTTGTTCC
in-rs9581943-Ot	ACTCACACCCCAAGTACCCGCGATCACCTGCTTTTGTTCC
fuel-rs9581943-Ef	CCCAAGTACCCGCGATCATCTGCTTTTGTTCCACCTACTC
fuel-rs9581943-Ot	CCCAAGTACCCGCGATCACCTGCTTTTGTTCCACTCACAC
rs9543325-Ef-M-top	GAGTGTGTACAGGTGTGATAGAGGTGCAGCAAGGGGATGTGT
rs9543325-Ef-M-btm	CCTTGCTGCACCTCTATCACACCTGTACACACTCCATCCTTAACTCCCTCAC
rs9543325-Ot-M-top	GTGGTTGAACAGGTGTGATAGAAGTGCAGCAAGGGGTGTTGA
rs9543325-Ot-M-btm	CCTTGCTGCACTTCTATCACACCTGTTCAACCACCATTACCAACCACCACTC
rs9543325-Ann-top	TCCATCCTTAACTCCCTCACGAGTGGTGGTTGGTAATGGTGGTTGA
rs9543325-Ann-btm	ACCATTACCAACCACCACTCGTGAGGGAGTTAAGGATGGAGTGTGT
rs9543325-Trans-top	TGTGTGAGGGAGTTAAGGATGGAGTGTGT
rs9543325-Trans-btm	ACTCCATCCTTAACTCCCTCACACACTATCCTAACTCACCACAC
in-rs9543325-Ef	ACACATCCCCTTGCTGCACCTCTATCACACCTGT
in-rs9543325-Ot	TCAACACCCCTTGCTGCACTTCTATCACACCTGT
fuel-rs9543325-Ef	CCTTGCTGCACCTCTATCACACCTGTACACACTC
fuel-rs9543325-Ot	CCTTGCTGCACTTCTATCACACCTGTTCAACCAC
rs7190458-Ef-M-top	GATGTGTGTGTGGGCGCTAAGGATGACGAACTTGATGTGTG
rs7190458-Ef-M-btm	AAGTTCGTCATCCTTAGCGCCCACACACACATCCACTACAACTCCACTCAC
rs7190458-Ot-M-top	GAGTTGAGGTGGGCGCTGAGGATGACGAACTTGTAGTGAG
rs7190458-Ot-M-btm	AAGTTCGTCATCCTCAGCGCCCACCTCAACTCCACAACCTACATCCAACC
rs7190458-Ann-top	TCCACTACAACTCCACTCACGGTTGGATGTAGGTTGTGGAGTTGAG
rs7190458-Ann-btm	TCCACAACCTACATCCAACCGTGAGTGGAGTTGTAGTGGATGTGTG
rs7190458-Trans-top	TGTGTGAGTGGAGTTGTAGTGGATGTGTG
rs7190458-Trans-btm	CATCCACTACAACTCCACTCACACACTATCCTAACTCACCACAC
in-rs7190458-Ef	CACACATCAAGTTCGTCATCCTTAGCGCCCACA
in-rs7190458-Ot	CTCACTACAAGTTCGTCATCCTCAGCGCCCAC
fuel-rs7190458-Ef	AAGTTCGTCATCCTTAGCGCCCACACACACATC
fuel-rs7190458-Ot	AAGTTCGTCATCCTCAGCGCCCACCTCAACTC

rs4795218-Ef-M-top	GAGTTGAGGAGTGGAAAATTGAGGATCGGAGAGGACAAGGGAGTAGAG
rs4795218-Ef-M-btm	CCTTGTCCTCTCCGATCCTCAATTTTCCACTCCTCAACTCCAACCAACCATTCCTCAC
rs4795218-Ot-M-top	GAGTGTAGGAGTGGAAAATTGAGGATCAGAGAGGACAAGGTGAGTGTAG
rs4795218-Ot-M-btm	ACCTTGTCCTCTCTGATCCTCAATTTTCCACTCCTACACTCCACTCCACAACTACACAC
rs4795218-Ann-top	TCCAACCAACCATTCCTCACGTGTGTAGTTGTGGAGTGGAGTGTAG
rs4795218-Ann-btm	TCCACTCCACAACTACACACGTGAGGAATGGTTGGTTGGAGTTGAG
rs4795218-Trans-top	TGTGTGAGGAATGGTTGGTTGGAGTTGAG
rs4795218-Trans-btm	ACTCCAACCAACCATTCCTCACACACTATCCTAACTCACCACAC
in-rs4795218-Ef	CTCTACTCCCTTGTCCTCTCCGATCCTCAATTTTCCACTC
in-rs4795218-Ot	CTACACTCACCTTGTCCTCTCTGATCCTCAATTTTCCACTC
fuel-rs4795218-Ef	CCTTGTCCTCTCCGATCCTCAATTTTCCACTCCTCAACTC
fuel-rs4795218-Ot	ACCTTGTCCTCTCTGATCCTCAATTTTCCACTCCTACACTC
rs7214041-Ef-M-top	GAGAAGTGTTAACAATATATTAGGAACTTATTTAGAGTGGATAGCTTAGAGGGAGAAGTG
rs7214041-Ef-M-btm	CCTCTAAGCTATCCACTCTAAATAAGTTCCTAATATATTGTTAACACTTCTCCACATTCACCCTACTCAC
rs7214041-Ot-M-top	GATGTGTGTTAACAATATATTAGGAACTTACTCAGATTGGATAGCTTAGAGATGTGTG
rs7214041-Ot-M-btm	TCCACTCCTATACTCCACCTACACACAATTGTTATATAATCCTTGAATGAGTCTAACCTATCGAATCT-5
rs7214041-Ann-top	TCCACATTCACCCTACTCACAGGTGAGGATATGAGGTGGATGTGTG
rs7214041-Ann-btm	TCCACCTCATATCCTCACCTGTGAGTAGGGTGAATGTGGAGAAGTG
rs7214041-Trans-top	TGTGTGAGTAGGGTGAATGTGGAGAAGTG
rs7214041-Trans-btm	TCTCCACATTCACCCTACTCACACACTATCCTAACTCACCACAC
in-rs7214041-Ef	CACTTCTCCCTCTAAGCTATCCACTCTAAATAAGTTCCTAATATATTGTTAA
in-rs7214041-Ot	CACACATCTCTAAGCTATCCAATCTGAGTAAGTTCCTAATATATTGTTAA
fuel-rs7214041-Ef	CCTCTAAGCTATCCACTCTAAATAAGTTCCTAATATATTGTTAACACTTCTC
fuel-rs7214041-Ot	TCTAAGCTATCCAATCTGAGTAAGTTCCTAATATATTGTTAACACACATC
rs1517037-Ef-M-top	GAGAAGAGTACAAACAAACAAAAAGCGAAAACAGAAAAATTGGAAACAGAGAAGAG
rs1517037-Ef-M-btm	TGTTTCCAATTTTTCTGTTTTCGCTTTTTGTTTGTTTGTACTCTTCTCCACTTCTCACCATCTCAC
rs1517037-Ot-M-top	GAGATGAGTACAAACAAACAAAAAGTGAAAACAGAAAAATTGGAAACAGAGATGAG
rs1517037-Ot-M-btm	TGTTTCCAATTTTTCTGTTTTCACTTTTTGTTTGTTTGTACTCATCTCCACTTCCCATACCACTAC
rs1517037-Ann-top	TCCACTTCTCACCATCTCACGTAGTGGTATGGGAAGTGGAGATGAG
rs1517037-Ann-btm	TCCACTTCCCATACCACTACGTGAGATGGTGAGAAGTGGAGAAGAG
rs1517037-Trans-top	TGTGTGAGATGGTGAGAAGTGGAGAAGAG
rs1517037-Trans-btm	TCTCCACTTCTCACCATCTCACACACTATCCTAACTCACCACAC

in-rs1517037-Ef	CTCTTCTCTGTTTCCAATTTTTCTGTTTTCGCTTTTTGTTTGTTTGTA
in-rs1517037-Ot	CTCATCTCTGTTTCCAATTTTTCTGTTTTCACTTTTTGTTTGTTTGTA
fuel-rs1517037-Ef	TGTTTCCAATTTTTCTGTTTTCGCTTTTTGTTTGTTTGTACTCTTCTC
fuel-rs1517037-Ot	TGTTTCCAATTTTTCTGTTTTCACTTTTTGTTTGTTTGTACTCATCTC
rs16986825-Ef-M-top	GAGAGAAGATTAGATTTTAACTATTTCCAGATTGTTCAAGCTATTAAGAATGTTGTA AAGTGAGAGAAG
rs16986825-Ef-M-btm	ACTTTACAACATTCTTAATAGCTTGAACAATCTGGAAATAGTTAAAATCTAATCTTC TCTCCAACCTTCTACCACTCAC
rs16986825-Ot-M-top	GTAGGATGATTAGATTTTAACTATTTCCAGATTGTTCGAGCTCTTAAGAATGTTGTA GTAGGATG
rs16986825-Ot-M-btm	TACAACATT CTTAAGAGCTCGAACAATCTGGAAATAGTTAAAATCTAATCATCCTA CCATCCCACATCACCAACT
rs16986825-Ann-top	TCCAACCTTCTACCACTCACAGTTGGTGATGTGGGATGGTAGGATG
rs16986825-Ann-btm	ACCATCCCACATCACCAACTGTGAGTGGTAGAAGGTTGGAGAGAAG
rs16986825-Trans-top	TGTGTGAGTGGTAGAAGGTTGGAGAGAAG
rs16986825-Trans-btm	TCTCCAACCTTCTACCACTCACACACTATCCTAACTCACCACAC
in-rs16986825-Ef	CTTCTCTCACTTTACAACATTCTTAATAGCTTGAACAATCTGGAAATAGTTAAAATC TAAT
in-rs16986825-Ot	CATCCTACTACAACATT CTTAAGAGCTCGAACAATCTGGAAATAGTTAAAATCTAA T
fuel-rs16986825-Ef	ACTTTACAACATTCTTAATAGCTTGAACAATCTGGAAATAGTTAAAATCTAATCTTC TCTC
fuel-rs16986825-Ot	TACAACATT CTTAAGAGCTCGAACAATCTGGAAATAGTTAAAATCTAATCATCCTA C
PRS-Thres-top	GTGTGGTGAGTTAGGATAG TGTGTGAG
PRS-Thres-btm	CTATCCTAACTCACCACAC
Highrisk-Res-top	GTAGA GTGTGGTGAGTTAGGATAG TGTGT
Highrisk-Res-btm	CTATCCTAACTCACCACAC TCTAC TCCTTCTACTCCACCTC
Res-fuel	CTATCCTAACTCACCACAC TCTAC
Repo-Res-top	ACTCCTTCTACTCCACCTC (3’-BHQ2)
Repo-Res-top	(5’-ROX) GAGGTGGAGTAGAAGGAGTAGAGTGT
cg16655791-Me-temp-forward	TTGGGGGCGTATTTACGTTACGGGTTTTGTTATTTTTATT**CG**GAAAATTTAAGTTGT TTGGGTATTAGGAGGGATTTTGA
cg16655791-Me-temp-reverse	TCAAAATCCCTCCTAATACCCAAACAACTTAAATTTTCCGAATAAAAATAACAAAAC CCGTAACGTAAATACGCCCCCAA
cg16655791-Um-temp-forward	TTGGGGGTGTATTTATGTTATGGGTTTTGTTATTTTTATTTGGAAAATTTAAGTTGT TTGGGTATTAGGAGGGATTTTGA
cg16655791-Um-temp-reverse	TCAAAATCCCTCCTAATACCCAAACAACTTAAATTTTCCAAATAAAAATAACAAAAC CCATAACATAAATACACCCCCAA
cg16655791-Me-MIP	AATAAAAATAACAAAACCCGACATCTCCTATCCAATCTCTCCTATCCAATCCTCATC TACAACCACTCATCTACAACCACTCATCTACAACCACTCATCTACAACCACATAAA CAACTTAAATTTTCCG
cg16655791-Um-MIP	AATAAAAATAACAAAACCCAATTCTACTAACTAACCACAACTATCCTCACCTAACAC TATCCTCACCTAACACTATCCTCACCTAACTCTAAACAACTTAAATTTTCCA

SI References

[1] C. A. Schneider, W. S. Rasband, K. W. Eliceiri, *Nat Methods* **2012**, *9* (7), 671.
[2] M. V. Relling, M. Schwab, M. Whirl-Carrillo, G. Suarez-Kurtz, C. H. Pui, C. M. Stein, A. M. Moyer, W. E. Evans, T. E. Klein, F. G. Antillon-Klussmann, K. E. Caudle, M. Kato, A. E. J. Yeoh, K. Schmiegelow, J. J. Yang, *Clin Pharmacol Ther* **2019**, *105* (5), 1095.
[3] Q. Ma, M. Zhang, C. Zhang, X. Teng, L. Yang, Y. Tian, J. Wang, D. Han, W. Tan, *Sci Adv* **2022**, *8* (47), eade0453.
[4] C. R. Paden, Y. Tao, K. Queen, J. Zhang, Y. Li, A. Uehara, S. Tong, *Emerg Infect Dis* **2020**, *26* (10), 2401.
